# Towards Acoustic Bioindicator Integration in AI-Based Wildfire Monitoring: A Systematic Review

**DOI:** 10.3390/s26185851

**Published:** 2026-09-15

**Authors:** Saba Mustafa, Mahsa Mohaghegh, Iman Ardekani, Abdolhossein Sarrafzadeh

**Affiliations:** 1 School of Engineering, Computing and Mathematical Sciences, Auckland University of Technology (AUT), Auckland 1010, New Zealand; saba.mustafa@autuni.ac.nz; 2 School of Arts & Sciences, The University of Notre Dame Australia, Sydney, NSW 2008, Australia; iman.ardekani@nd.edu.au; 3 Batten College of Engineering and Technology, Old Dominion University, Norfolk, VA 23529, USA; asarrafz@odu.edu

**Keywords:** wildfire monitoring, early warning systems, remote sensing, Internet of Things (IoT), machine learning, multimodal sensing, bioindicators, bee bioacoustics

## Abstract

Wildfires are becoming more frequent, severe, and long-lasting, driving rapid growth in sensor-based and artificial intelligence (AI)-enabled systems for early detection and risk assessment. This article presents a systematic literature review, based on 169 studies screened from 7511 records, of wildfire monitoring approaches using satellite and aerial remote sensing, fixed cameras, wireless sensor networks, and Internet of Things (IoT) platforms combined with machine learning (ML) and deep learning (DL) models for ignition detection, fire-weather indices, spread prediction, and burned-area mapping. The review organizes existing work by sensing modality, spatial and temporal scale, learning task, model type, and deployment architecture, and identifies the environmental drivers most commonly used across systems, including temperature, humidity, vegetation state, drought indices, and smoke or air quality. Based on this analysis, the review summarizes key technical challenges, including data sparsity in remote regions, high false-alarm rates, limited edge resources, and difficulty fusing heterogeneous data streams in real time. As an exploratory future direction, the review discusses bioindicator signals from wildlife and managed species, using honeybee colonies as a case example. Current bee bioacoustic studies support the detection of colony states and environmental stress proxies, but they do not yet validate wildfire or smoke detection. Therefore, this review proposes bee bioacoustics only as a potential complementary contextual signal for future hybrid wildfire monitoring systems.

## 1. Introduction

**Figure 1:**
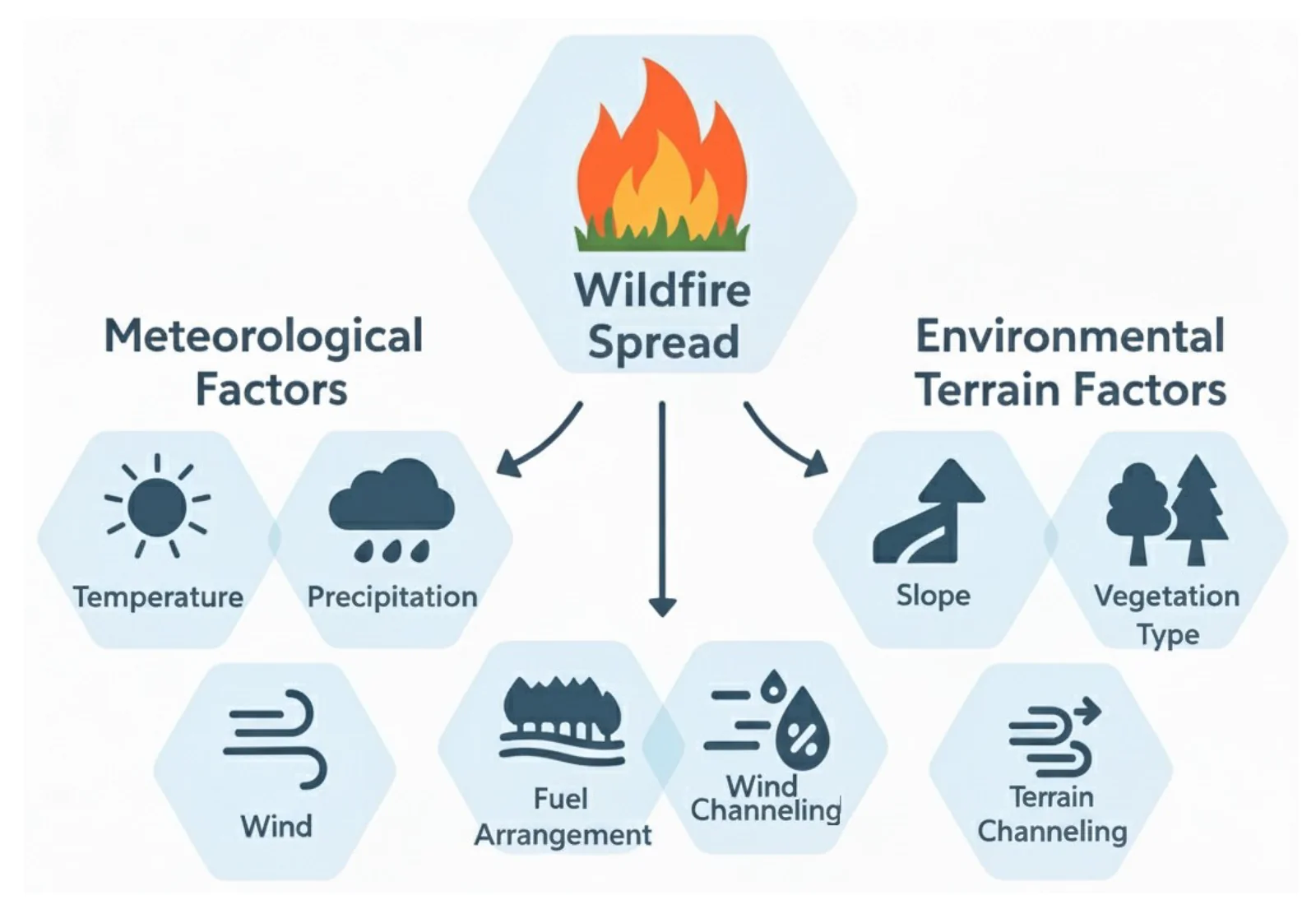
Conceptual diagram of meteorological and environmental terrain factors influencing wildfire spread, including temperature, precipitation, wind, fuel arrangement, wind channeling, slope, vegetation type, and terrain channeling.

Wildfires are increasingly manifesting with heightened frequency, intensity, and unpredictability across various global regions, attributable to a confluence of climate change, modified land-use practices, and fuel accumulation [1,2,3]. Significant “megafire” incidents, exemplified by the 2019–2020 Australian bushfires and recent fire seasons in North America and the Mediterranean, have resulted in considerable fatalities, infrastructural damage, and loss of ecosystem services, while also emitting substantial volumes of greenhouse gases and aerosols into the atmosphere [4,5,6]. Traditional detection methods based on lookout towers, manual patrols, and limited sensor networks are often slow, labor-intensive, and constrained by line-of-sight and weather conditions, leading to delayed responses and higher suppression costs [7,8,9]. In this context, there is a compelling need for scalable, dependable, and near-real-time systems capable of early ignition detection, fire spread tracking, and providing robust indicators of heightened fire risk prior to ignition [10,11]. Figure 1 summarizes the key meteorological and terrain factors that influence wildfire spread, including temperature, precipitation, wind, fuel arrangement, wind channeling, slope, vegetation type, and terrain channeling, which jointly determine ignition likelihood and fire behavior [5,6,12].

Wildfire detection research spans several sensing domains, including satellite and aerial remote sensing, UAV-based imaging, ground-based IoT and wireless sensor networks, and specialized local systems, each contributing complementary spatial and temporal information [13,14,15,16,17,18]. Across these domains, artificial intelligence, particularly machine learning and deep learning, plays a central role in automatic detection, segmentation, and risk prediction, using methods that range from classical models such as random forests and gradient-boosted trees to CNN-, encoder–decoder-, and transformer-based architectures for pixel-level fire and smoke segmentation and object detection [19,20,21,22]. Section 1.2 discusses these sensing domains and AI methods in more detail, together with their respective strengths and limitations.

Despite these advances, several cross-cutting limitations remain across the literature. Many AI models are trained on region-specific datasets and struggle to generalize across ecosystems, sensor types, and fire regimes, particularly in data-scarce regions or under extreme conditions [23,24,25]. Class imbalance, annotation scarcity, and the rarity of early-stage ignition samples hinder robust detection and increase false alarm rates [13,26]. IoT and UAV-based systems must operate under tight energy, communication, and computational constraints, making it challenging to deploy complex DL models at the edge while maintaining real-time performance and reliability [27,28,29]. Moreover, most existing systems rely exclusively on physical and meteorological variables, with limited exploitation of biological or ecological signals that may respond sensitively to pre-ignition environmental stressors such as heat, humidity changes, and air pollution [30]. In parallel, research in fire ecology and wildlife biology has shown that fire and wildfire smoke can affect animal behavior, demography, and health across multiple taxa, indicating that many species respond sensitively to fire-related disturbances and smoke exposure [31,32,33,34].

Nature-inspired sensing provides a complementary perspective for future wildfire monitoring research. Bioindicator species, including insects and birds, can respond to environmental change and have been used to indicate ecosystem condition [35,36]. Studies show that bee behavior and acoustic characteristics can vary with environmental conditions and stressors, and that these signals can be analyzed using machine learning methods [30,37]. Separate ecological evidence also shows that bees can respond behaviorally to fire disturbance [38]. However, the available bee-monitoring literature does not yet validate bee acoustic signals for direct wildfire detection, wildfire-smoke event classification, or ignition localization. Existing studies mainly classify colony states, health conditions, acoustic patterns, or environmental stress proxies rather than wildfire-related events. Bee responses may also be affected by weather, colony state, disease, forage availability, management activity, and other local conditions. Therefore, bee bioacoustics should not be considered a stand-alone wildfire detection method. Instead, it should be considered an exploratory contextual sensing layer that may support future hybrid systems when combined with georeferenced physical sensors, weather data, air-quality measurements, cameras, thermal sensing, or UAV observations. Section 4 discusses this hypothesis and the validation studies needed before operational use.

In this context, the present article provides a systematic literature review of AI-enabled wildfire detection and early risk indication across sensing domains. The review is guided by three research questions:

- **RQ1 (Domain level—technologies and data):** What major sensing and data domains (satellite and aerial remote sensing, UAV imaging, IoT/WSN ground sensing, and local or specialized systems) have been used for wildfire detection and early risk indication since 2019, and what types of environmental variables and spatio-temporal scales do they each capture?
- **RQ2 (AI methods across domains):** How have AI methods (machine learning and deep learning) been applied within these domains for wildfire detection, segmentation, and risk prediction, and how do their reported performance metrics compare across different datasets, ecosystems, and tasks (e.g., classification, time series forecasting, and spatio-temporal prediction)?
- **RQ3 (Generalization, integration and uncertainty):** To what extent do current AI-based wildfire systems generalize across regions, sensors, and fire regimes, and how do studies address key challenges such as data scarcity, multimodal data integration, real-time constraints, and uncertainty quantification?

Building on the systematic synthesis, this article also presents a forward-looking discussion of bioindicator and nature-inspired sensing approaches, including bee bioacoustics. This discussion is not a formally screened research question and does not claim that bee signals currently improve wildfire detection performance. Instead, it considers whether bee-derived acoustic and environmental anomalies could be evaluated in future as complementary contextual information within conventional AI-based wildfire monitoring systems.

### 1.1. Contribution of This Paper

The main contributions of this article are threefold. First, it proposes a domain-oriented taxonomy of wildfire detection research that unifies satellite/aerial, UAV, IoT/WSN, and local/specialized systems, and maps them to the environmental variables and scales they observe (RQ1). Second, it provides a structured synthesis of AI methods across these domains, including detailed tables of ML/DL architectures, datasets, and performance metrics, and identifies patterns in how different models perform under varying sensing conditions (RQ2–RQ3). Third, drawing on supporting evidence from bee bioacoustics and broader bioindicator research, the article outlines a conceptual hybrid sensing and monitoring framework. In this framework, bee-derived acoustic signals are treated as a potential contextual or anomaly-based input rather than as a direct wildfire detector. Current evidence supports AI-based analysis of colony states and environmental stress proxies, but does not yet demonstrate wildfire detection, wildfire-smoke event classification, or ignition localization. The proposed role of bee-derived signals is therefore to support the prioritization or verification of possible local environmental anomalies when combined with georeferenced physical sensors and other conventional wildfire-monitoring data. Accordingly, the bioindicator component is presented as an exploratory future research direction, not as a co-equal systematically reviewed sensing domain.

### 1.2. Related Work

Recent progress in wildfire monitoring has been driven by advances in sensing, machine learning, and multimodal data integration. In wildfire research, satellite and aerial remote sensing have been widely used to identify hotspots, map burned areas, and assess fire-related environmental impacts, while UAVs have enabled higher-resolution fire and smoke monitoring at local scales [13,39,40,41,42,43,44]. Ground-based IoT and wireless sensor network systems have also been used to monitor temperature, humidity, gas concentrations, and other environmental variables for early warning and local detection [16,29,45,46,47]. In addition, specialized approaches have combined CCTV, audio sensing, meteorological information, and social media streams to complement physical sensing and improve situational awareness [17,18,48,49].

Across these domains, artificial intelligence has become central to wildfire detection, segmentation, and prediction. Classical machine learning models have been used for fire occurrence prediction and risk mapping, while CNNs, encoder–decoder architectures, and transformer-based models have improved pixel-level fire and smoke segmentation from satellite, UAV, and ground-camera imagery [19,21,50,51,52,53,54,55,56,57,58,59]. More recent multimodal and transformer-enhanced approaches have further explored the fusion of satellite, meteorological, UAV, and sensor data to improve detection and forecasting performance [60,61,62,63,64,65,66,67]. However, many of these systems remain limited by region-specific training data, class imbalance, sparse early-ignition samples, and difficulties in real-time deployment under edge constraints [27,68,69,70,71,72].

A smaller but emerging body of research examines biological and ecological signals as complementary sources of environmental information. Studies in fire ecology and wildlife biology show that fire and wildfire smoke can affect animal behavior, movement, health, and disease patterns [31,34,73]. Bioindicator species, including insects and birds, have also been proposed as indicators of ecosystem change because they can reflect environmental disturbance over time [35,36]. Studies of honeybee colonies show that temperature, humidity, air quality, and colony condition can affect behavior and hive activity [74,75,76]. Hive acoustic signals can also change under environmental disturbance and can be used to identify colony-related conditions [77,78]. Machine learning methods have been applied to bee acoustic data for automated classification and monitoring [30,37,78].

Based on this background, the present review systematically synthesizes AI-based wildfire sensing, detection, prediction, and monitoring studies. It separately discusses bee bioacoustics and bioindicator-based sensing as an exploratory future research direction. The purpose is not to present bees as a validated wildfire sensing modality. Instead, the discussion examines how hive acoustic and environmental data could be collected alongside physical wildfire measurements in future field studies to test whether they add useful contextual information to established monitoring systems.

### 1.3. Outline of This Paper

The remainder of this article is organized as follows. Section 2 describes the systematic review methodology, including database selection, search strategy, eligibility criteria, screening procedure, and quality assessment. Section 3 presents the results and findings, beginning with the taxonomy of wildfire monitoring technologies and sensing modalities, followed by AI methods across wildfire sensing domains and cross-domain challenges. Section 4 discusses open challenges and outlines bioindicator-based sensing, including bee bioacoustics, as a forward-looking direction for future hybrid wildfire monitoring systems. Finally, Section 5 concludes the paper.

## 2. Methodology

The SLR is guided by three research questions (RQ1–RQ3) that focus on sensing domains, AI methods, and generalization and integration challenges, as stated in the Introduction. These questions define the scope of the search protocol, eligibility criteria, and subsequent analysis.

### 2.1. Database Selection

Two primary electronic databases were selected: Scopus and IEEE Xplore. Scopus, maintained by Elsevier, is a large abstract and citation database with broad coverage of environmental sciences, engineering, technology, computer science, and related disciplines, and therefore is well suited to capture research on remote sensing, UAV imaging, and IoT/WSN systems. IEEE Xplore focuses on scientific and technical content in electrical engineering, computer science, and electronics and was used to retrieve work on AI algorithms, embedded systems, IoT architectures, and signal-processing techniques relevant to wildfire monitoring. This combination follows recommendations for systematic reviews that pair a multidisciplinary citation database with subject-specific databases to broaden coverage and minimize selection bias.

### 2.2. Search Strategy and Keywords

We searched Scopus and IEEE Xplore in titles, abstracts, and keywords using combinations of wildfire terms with detection, prediction, and monitoring terms plus AI, remote-sensing, and IoT-related keywords. The final Boolean expressions and initial record counts are reported in Table 1. The search combined wildfire-related terms with detection/monitoring terms and AI/sensing terms, further refined using Scopus exact-keyword filters (e.g., Remote Sensing, Wildfire, Machine Learning, Forest Fires) and restricted to publications between 2019 and 2025.

### 2.3. Inclusion and Exclusion Criteria

Unified inclusion and exclusion criteria were applied to ensure consistency across the review. These criteria helped retain studies directly relevant to wildfire sensing and AI-based analysis.

Table 2 presents the eligibility criteria for study selection.

### 2.4. Screening Process and PRISMA Flow

All retrieved records were exported to a reference management tool, where duplicates across Scopus and IEEE Xplore were removed. The remaining records were screened in three stages: (i) title screening, (ii) abstract screening, and (iii) full-text assessment. Two reviewers independently screened titles and abstracts against the inclusion criteria; disagreements were resolved through discussion and, where necessary, consultation with a third reviewer.

Full texts that passed the abstract screening were then assessed for eligibility against the detailed criteria in Section 2.3. Typical reasons for exclusion at this stage included a lack of wildfire focus, the absence of AI/ML or sensing content, insufficient methodological detail, or purely post-fire analysis.

**Figure 2:**
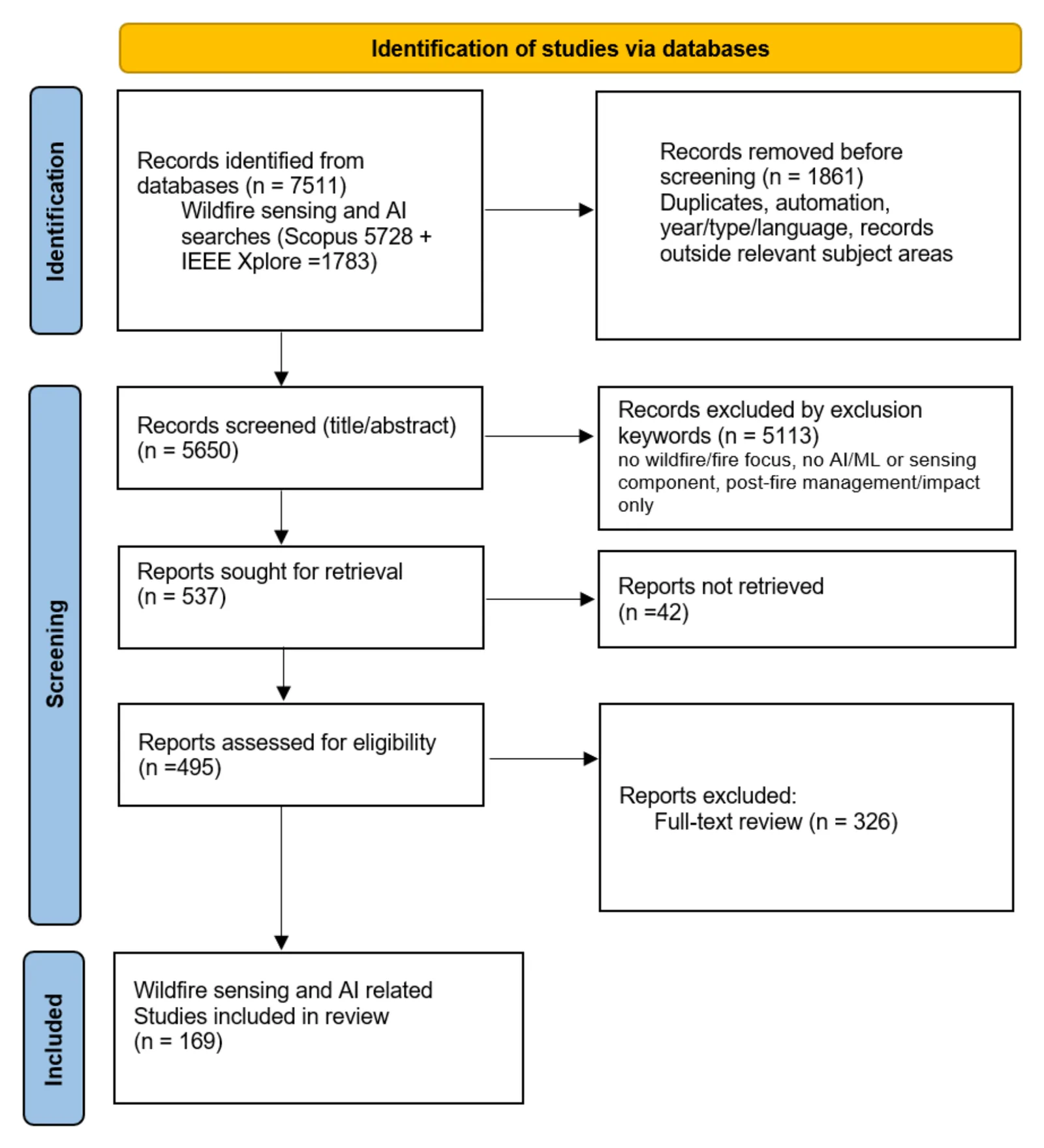
PRISMA 2020 flow diagram for the systematic review of wildfire sensing and AI studies, summarizing the identification, screening, and inclusion process, resulting in 180 included studies.

This systematic review was conducted and reported in accordance with the Preferred Reporting Items for Systematic Reviews and Meta-Analyses (PRISMA) 2020 guidelines [79]. The study selection process followed the PRISMA guidelines and is summarized in Figure 2. A total of 7511 records were identified from database searches in Scopus (*n* = 5728) and IEEE Xplore (*n* = 1783) for the wildfire sensing and AI stream. Before screening, 1861 records were removed as duplicates, by automation, or because they did not meet the basic publication year, document type, or language criteria, leaving 5650 records for title and abstract screening. After title and abstract screening, 5113 records were excluded using predefined exclusion criteria, including a lack of wildfire/fire focus, absence of an AI/ML or sensing component, and studies limited to post-fire management or impact, leaving 537 reports sought for retrieval. Of these, 42 reports could not be retrieved despite institutional and inter-library access attempts, leaving 495 reports assessed for eligibility. At the full-text stage, 326 reports were excluded because they did not meet the review eligibility criteria. The main reasons were that they did not focus on wildfire monitoring or detection, did not use AI, ML, sensing, or another relevant analytical method, focused only on post-fire impacts or management, or did not provide enough methodological detail. Some reports met more than one exclusion criterion. We did not retain separate counts for each exclusion reason during screening; therefore, the reasons are described here without separate numerical totals. In total, 169 studies met all inclusion criteria and were retained for qualitative synthesis.

In addition, selected studies on fire–animal interactions, wildlife responses to wildfire smoke, bee bioacoustics, hive monitoring, and ecological bioindicators were consulted to inform the exploratory discussion in Section 4. These studies were not included in the PRISMA-guided systematic corpus because the review eligibility criteria were restricted to AI-based wildfire sensing, detection, prediction, and monitoring studies.

The contextual literature was identified through targeted background searching and reference checking using terms related to honeybee acoustics, hive monitoring, environmental stress, air pollution, smoke exposure, bioindicators, and animal responses to fire. This search was intended to support a conceptual future-direction discussion rather than to form a separate systematic review. Therefore, these sources are not included in the PRISMA flow diagram (Figure 2), quality assessment, or quantitative synthesis of the 169 included wildfire-monitoring studies.

### 2.5. Data Extraction

Data were extracted independently by two reviewers using a standardized template, piloted on 15 studies before full extraction began. Disagreements were resolved through discussion between the two reviewers. For each study, we recorded: study details (authors, year, venue, country); sensing domain (satellite, UAV, IoT/WSN, or local systems); AI/ML method used; task type (detection, segmentation, or prediction); dataset characteristics (source, size, coverage); environmental input variables (e.g., temperature, humidity, vegetation, smoke); reported performance metrics (e.g., accuracy, F1-score); and deployment constraints (latency, energy, computation), where reported. As this review focuses on technical characteristics rather than clinical outcomes, no formal outcome pre-specification was applied. Missing or unclear data were recorded as “not reported” rather than estimated, and relevant gaps are discussed in Section 3. A formal meta-analysis was not conducted because the included studies addressed different tasks, such as fire and smoke classification, object detection, segmentation, risk prediction, fire-spread forecasting, and edge deployment. They also used different measures, including accuracy, F1-score, mAP, IoU, RMSE, latency, and energy use. The studies used different datasets, sensors, regions, spatial resolutions, and validation methods. Therefore, combining their results into one cross-domain performance value would not provide a meaningful comparison. Instead, the findings were compared by sensing domain, task type, model family, dataset context, reported metric, and deployment setting.

The review was registered retrospectively with the Open Science Framework (OSF) after the manuscript was completed and submitted. The registration DOI is https://doi.org/10.17605/OSF.IO/VH7KE. No prospective protocol was publicly available before the review began. The authors defined the review questions, eligibility criteria, search strategy, and data-extraction approach before screening and synthesis. Retrospective registration is a limitation because the protocol was not publicly available before the review process. However, the review methods are reported transparently in this manuscript.

### 2.6. Quality Assessment

In addition to eligibility screening, a lightweight quality assessment was conducted to evaluate the methodological rigor and completeness of reporting in the included studies, following practices recommended in recent systematic reviews of AI- and IoT-enabled systems. Each study was assessed against five criteria tailored to this review: (i) problem definition and context (clear statement of objectives, wildfire scenario and sensing domain); (ii) data description (adequate description of datasets, including sources, size, spatial/temporal coverage and class balance); (iii) methodological detail and reproducibility (sufficient information on pre-processing, feature extraction, model architecture and training procedure); (iv) evaluation protocol and metrics (use of appropriate metrics, validation strategy such as cross validation or spatial/temporal train–test separation, and comparison with baselines); and (v) limitations and threats to validity (explicit discussion of limitations, generalization issues or deployment constraints).

Each criterion was rated on a three-point scale (0 = not addressed, 1 = partially addressed, and 2 = clearly addressed), and the average scores are reported in Table 3. The five criterion ratings (QA1–QA5) were summed to produce an overall score for each study. The score was not used as an automatic exclusion criterion because lower-scoring studies could still provide useful information about specific sensing setups, deployment settings, or under-studied regions. However, studies with lower scores were given less weight in the narrative synthesis. Their results were used mainly to describe the range of approaches in the field, rather than to support strong conclusions about model performance, generalization, or operational readiness. In contrast, conclusions about performance, validation, and real-world deployment were based mainly on studies with clearer data descriptions, methodological details, evaluation procedures, and discussion of limitations. The quality scores were not used for statistical meta-analysis.

To provide an overall view of reporting quality, the five QA scores were summed for each included study, giving a total score from 0 to 10. Studies were grouped as low reporting quality (0–4), moderate reporting quality (5–7), or high reporting quality (8–10). Of the 169 included studies, approximately 2% were classified as low reporting quality, 38% as moderate reporting quality, and 60% as high reporting quality. These quality bands were used to inform the interpretation and weighting of evidence in the narrative synthesis, rather than as additional exclusion criteria.

Overall, most studies clearly state their objectives and provide sufficient methodological detail for approximate replication (QA1 ≈ 1.74; QA3 ≈ 1.73), whereas dataset descriptions and evaluation protocols are more variable (QA2 ≈ 1.61; QA4 ≈ 1.60). QA5, which assessed explicit discussion of limitations and threats to validity, received the lowest mean score (1.34/2). This indicates that many studies did not fully report generalization risks, deployment constraints, or threats to operational validity. Therefore, reported performance on local datasets should not be interpreted as evidence of reliable cross-region, cross-sensor, or real-world deployment readiness without stronger validation.

## 3. Results and Findings

This section presents a structured review of wildfire detection techniques reported in the literature, focusing on how different sensing modalities and data sources are integrated with artificial intelligence (AI) methods. Rather than organizing studies solely by algorithm type, the review adopts a domain-oriented perspective that reflects where and how sensing takes place, including satellite and aerial platforms, unmanned aerial vehicles (UAVs), ground-based IoT and wireless sensor networks (WSNs), and local or specialized monitoring systems. This perspective enables a clearer understanding of how data characteristics, spatial and temporal scales, and environmental variables influence the design and performance of AI-based wildfire detection systems.

**Quantitative overview of included studies.** The 169 included studies covered multiple sensing and modeling categories, and individual studies could contribute to more than one category. Among studies assigned to the four principal sensing domains, satellite and aerial remote sensing formed the largest group, represented in approximately 55 studies (30.6%), followed by local or specialized sensing systems in 35 (19.4%), IoT/WSN-based ground sensing in 33 (18.3%), and UAV-based sensing in 25 (13.9%). The remaining studies addressed cross-cutting architectures, such as federated or distributed learning, or could not be assigned to one principal sensing domain because they combined multiple modalities.

In terms of analytical tasks, fire or smoke detection and monitoring was the dominant application, addressed in approximately 117 studies (65.0%). Risk prediction or forecasting was considered in 32 studies (17.8%), segmentation or burned-area mapping in 15 (8.3%), and multimodal or hybrid sensing/model architectures in 17 (9.4%). Across model families, convolutional neural networks and YOLO-based detectors were the most frequently reported deep learning approaches, particularly for image-, UAV-, and satellite-based fire or smoke detection, whereas classical machine learning methods were more commonly applied to structured meteorological, environmental, and ground-sensor data. Because individual studies could involve more than one task or model family, the task and model-family categories are not mutually exclusive; therefore, their percentages should not be interpreted as summing to 100%.

### 3.1. Sensing Modalities for Wildfire Detection (RQ1)

To answer RQ1, we grouped the included AI-enabled wildfire studies into a domain-level taxonomy that reflects where and how sensing takes place, rather than classifying systems purely by algorithm type. Drawing on recent reviews that distinguish satellite, UAV, and ground-based architectures while emphasizing their increasing AI integration, we identify four principal technical domains: satellite and aerial remote sensing, UAV-based detection, IoT/WSN ground sensing, and local/specialized systems.

**Figure 3:**
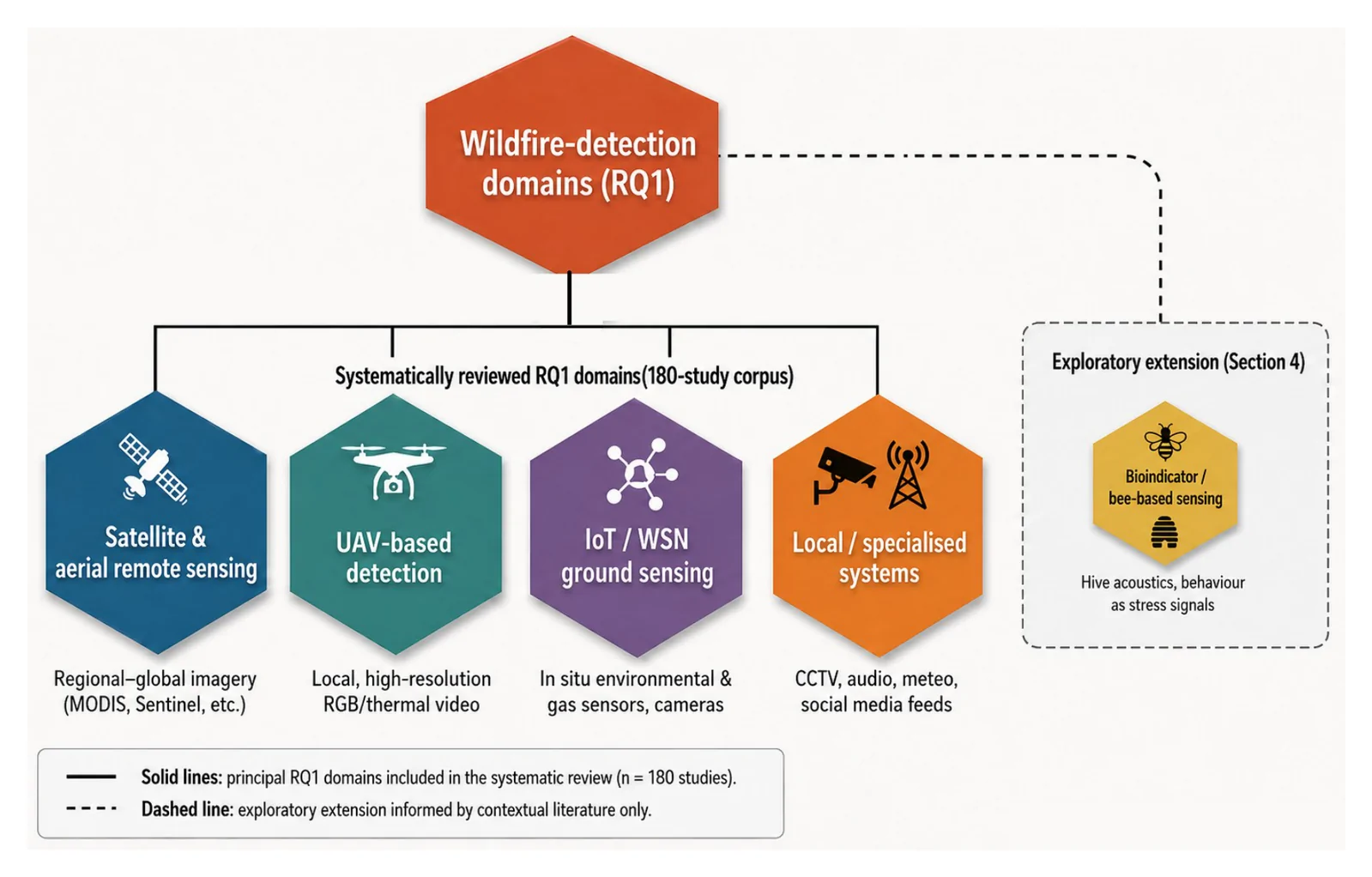
Taxonomy of wildfire detection domains (RQ1) represented as a hexagon-based tree. A central node for “wildfire detection domains (RQ1)” branches into the four main technical domains identified in this review.

Figure 3 presents the four technical sensing domains included in the PRISMA-guided systematic review: satellite and aerial remote sensing, UAV-based detection, IoT/WSN ground sensing, and local or specialized systems. These four domains represent the 169-study systematic corpus. Bioindicator and bee-based sensing is shown separately as an exploratory future direction informed by the contextual literature. It was not included as a fifth technical domain because it was not part of the PRISMA-guided corpus. The dashed connection in Figure 3 shows potential future hybrid integration that requires field validation. Section 3.1.1, Section 3.1.2, Section 3.1.3 and Section 3.1.4 describe the four reviewed domains, including their data types, spatial and temporal scales, key variables, strengths, limitations, and suitable wildfire-monitoring tasks.

The following subsections elaborate each domain in turn, examining the characteristic sensing setups, the roles played by machine learning and deep learning techniques, and the main strengths and limitations that motivate hybrid, multimodal architectures.

#### 3.1.1. Satellite and Aerial Remote Sensing

Satellite and aerial remote sensing provides wide-area and repeatable observations for wildfire monitoring. These systems use multispectral, thermal, and hyperspectral imagery to detect active fires, map burned areas, and assess fire-related environmental effects. Satellite data can also support emissions and fire-intensity analysis. Gupta et al. [80] used satellite data to assess stubble-burning emissions. Overall, this sensing domain is most useful for regional surveillance, hotspot mapping, burned-area mapping, fire-risk estimation, and fire-spread monitoring [39,40,80].

Across the reviewed literature, satellite imagery supports three main task families: active-fire detection, burned-area mapping, and environmental or emissions analysis. For active-fire detection, studies such as those by Saul’skii [81], Xu and Zhong [82], Kang et al. [83], and Wang et al. [72] show how geostationary and polar-orbiting satellite data can be used to identify thermal anomalies and track fire development over time. For burned-area mapping, Hu et al. [20], Zhang et al. [84], and Seydi and Sadegh [85] demonstrate the value of deep learning and multi-sensor fusion for delineating fire scars and post-fire change. In parallel, studies on fire radiative power and related variables, including Dong et al. [86], Thornberry et al. [87], and Mazzeo et al. [88], indicate that satellite-based products can also support quantitative analysis of fire intensity and spread. These works collectively show that AI-enabled remote sensing is increasingly used not only to detect fires, but also to characterize their dynamics and ecological consequences [20,86,88].

The reviewed studies show three main task types: active-fire detection, burned-area mapping, and fire-intensity or emissions analysis. Active-fire detection commonly uses thermal anomalies from geostationary or polar-orbiting satellites. Burned-area mapping usually uses multi-temporal satellite imagery with machine learning or deep learning models. Fire radiative power and related satellite products can provide information about fire intensity and fire development over time [20,81,86,87,88].

A clear trend is the use of machine learning and deep learning instead of simple threshold-based methods. CNN-based, segmentation, and multi-sensor models can use spectral and spatial information to improve fire boundary mapping and smoke or fire detection. Ali and Kurnaz [21] show that deep learning methods can improve fire delineation. Temporal models can also combine satellite data with contextual environmental information for detection and monitoring tasks [54,57,65,89].

However, model performance depends strongly on image quality, sensor type, spatial resolution, atmospheric conditions, class balance, and the availability of labeled fire data. Many studies report good results on local datasets, but fewer test whether models work across different ecosystems, sensors, regions, and fire regimes. Therefore, strong performance on one dataset should not be treated as evidence of broad operational reliability [50,65,89].

Satellite and aerial systems are a strong foundation for regional and large-area wildfire monitoring. However, cloud and smoke occlusion, mixed pixels, revisit delays, and low sensitivity to small or short-duration fires can limit very early ignition detection [39,40,83]. For this reason, satellite and aerial data are most effective when combined with higher-resolution UAV sensing, ground-based IoT/WSN systems, and local monitoring systems [42,82,90].

#### 3.1.2. UAV-Based Detection

UAV-based systems use rotary- or fixed-wing drones with RGB, thermal, multispectral, or infrared sensors. They provide high-resolution observations of fire-prone areas, smoke plumes, active fire fronts, and post-fire conditions. Compared with satellite systems, UAVs can collect detailed local data, operate below cloud cover, and be deployed when needed. Therefore, they are useful for local surveillance, hotspot localization, fire-front monitoring, and tactical support [91,92].

UAV imagery is mainly used for smoke and flame detection, segmentation, early warning, and fire-front tracking. Deep learning models are common because they can analyze complex visual data. CNN-based and YOLO-based models are widely used for fast fire and smoke detection in RGB, thermal, and infrared images. Mukhiddinov et al. [55] show that YOLO-based models can support smoke and fire detection in UAV imagery. Lightweight models are especially important because UAVs have limited onboard computing power, battery capacity, and communication bandwidth [52,93,94,95,96].

A second line of work extends UAV monitoring toward multimodal fusion, swarm coordination, and early risk prediction. Niu et al. [97] and Qiao et al. [98] show that combining visible and infrared imagery can improve early fire detection and enable additional tasks such as distance estimation, while Zhu et al. [22] and Yandouzi et al. [99] illustrate the benefits of multi-source fusion and semantic segmentation for more robust fire localization. In these systems, UAVs can provide close-range visual verification after an environmental sensor or satellite system indicates a possible fire event [22,97,98,99,100,101,102]. Trajectory planning and swarm coordination can further improve coverage, but they add communication, safety, and energy-management requirements [103,104,105].

UAVs are therefore a bridging layer between regional satellite monitoring and local ground sensing. They are most useful for rapid visual confirmation, local smoke and flame detection, hotspot localization, and detailed monitoring of active fire fronts. However, their use is limited by flight endurance, payload capacity, weather sensitivity, communication range, regulations, and safe navigation in fire conditions [15,92,106,107].

UAV systems should not be treated as a stand-alone solution for large or long-duration wildfire events. Their strongest role is within a multimodal wildfire-monitoring system. Satellite and fire-weather data can provide regional context, IoT/WSN sensors can provide continuous local monitoring, and UAVs can provide targeted visual verification and high-resolution observations [100,108,109].

#### 3.1.3. IoT and WSN Ground Sensing

IoT and wireless sensor network (WSN) systems use distributed sensors in fire-prone areas. These sensors commonly measure temperature, humidity, gas, smoke, wind, and light. They provide continuous local monitoring and can detect environmental changes before flames become visible to satellite or aerial platforms. Therefore, IoT/WSN systems are useful for local early warning, environmental anomaly detection, fire-weather monitoring, and sensor-triggered alerts [45,110,111].

Compared with satellite and UAV systems, IoT/WSN systems provide more frequent measurements, often at seconds-to-minutes intervals. However, they cover only a limited area. Their performance depends on sensor placement, node density, communication reliability, power management, and maintenance [45,112].

The reviewed studies mainly use machine learning and deep learning for anomaly detection, risk prediction, sensor-data classification, and multi-sensor fusion. These models use environmental data to distinguish normal weather variation from patterns linked to possible ignition or rapid fire spread. Benzekri et al. [16] show that sensor data and classification methods can support automated wildfire alerts. Edge and fog computing can reduce latency and communication needs by processing sensor data near the source [27,28,53].

A key finding is that the main challenge is not only detection accuracy. IoT/WSN systems must also operate for long periods in remote and harsh environments. Energy-efficient communication, routing, sleep scheduling, solar power, low-power hardware, and fault tolerance are important for reliable operation [46,113,114,115,116,117,118,119].

IoT/WSN systems are increasingly used as part of hybrid wildfire-monitoring systems. Ground sensors can trigger UAV checks, send data to edge or cloud systems, and combine with satellite, weather, and camera data. This can improve local awareness and reduce the time needed for verification [100,101,120,121,122,123]. Fire-danger models also increasingly combine sensor data with weather and environmental indices instead of using simple fixed thresholds [63,111].

Overall, IoT/WSN ground sensing is most suitable for continuous local early warning in high-risk and instrumented areas. It complements satellite and UAV systems by providing dense, low-latency measurements near possible ignition zones. However, its operational value depends on reliable networking, energy-aware design, good sensor placement, and integration with other sensing systems [10,45].

Many IoT/WSN studies use prototype systems, simulations, or single-site data. Therefore, strong results may not transfer directly to other landscapes, sensor layouts, weather conditions, communication environments, or fire regimes.

#### 3.1.4. Local and Specialized Systems

Local and specialized systems are designed for a specific site, landscape, infrastructure, or management workflow. They do not provide the wide coverage of satellite systems or the mobility of UAVs. Instead, they use fixed sensors and local communication systems. Common inputs include CCTV cameras, weather stations, air-quality sensors, acoustic devices, and local alert services. These systems support continuous site-specific monitoring and operational decision support [124,125].

Local systems commonly combine climate, weather, fuel, and vegetation information with sensor data. These variables help estimate fire risk and provide context for local monitoring. Bowman [124] states that effective wildfire monitoring should connect local observations with regional climate drivers, fuel conditions, and fire regimes. This approach can support early-warning dashboards and local decision-support systems [1,5,12,17,125,126,127,128,129].

Air-quality monitoring is also important in local systems. Fixed stations can measure smoke-related pollutants and other environmental changes near fire-prone areas. Muthukumar et al. [130] show that machine learning models can predict PM_2.5_ using wildfire and weather variables. Such systems can support local air-quality monitoring, fire-impact assessment, and public-health response, in addition to fire detection [130,131,132,133,134].

Local systems also provide data for AI-based fire and smoke detection. Fixed cameras and embedded devices can support continuous smoke and flame detection at a specific location. These systems often use CNN-based or lightweight detection models because they need to operate with limited computing resources. Wang et al. [72] provide an open-flame and smoke dataset that can support the training and evaluation of camera-based detection models. Local systems can also combine camera data, weather data, air-quality measurements, and alert services to support automated decision-making [17,49,135].

Audio sensing can provide an additional local signal when visual data are unclear. Huang et al. [18] show that embedded audio systems can detect wildfire-related sound patterns on resource-constrained hardware. Acoustic data should be used as complementary evidence because wind, background noise, distance, and terrain can affect the signal. More broadly, local systems can combine cameras, weather stations, air-quality sensors, acoustic devices, and communication services within a multimodal monitoring system [124,125,136].

Overall, local and specialized systems are most suitable for continuous monitoring, fixed-camera smoke and flame detection, local air-quality monitoring, embedded audio detection, and multi-source alerting. Their main limitations are limited field of view, site-specific calibration, infrastructure needs, communication reliability, and maintenance requirements. Many systems are tested at one or a few sites. Therefore, their performance may not transfer directly to other locations, landscapes, weather conditions, camera positions, or fire regimes.

#### 3.1.5. Cross-Domain Synthesis of Sensing Modalities

Table 4 synthesizes the domain-specific evidence presented in Section 3.1.1, Section 3.1.2, Section 3.1.3 and Section 3.1.4 by comparing the operational roles, strengths, limitations, and suitable wildfire-monitoring tasks of the four sensing domains.

The synthesis shows that no single sensing domain is suitable for every wildfire-monitoring task. Satellite and aerial systems provide regional context, IoT and WSN systems provide continuous local monitoring, and UAVs or local systems support high-resolution verification. These complementary roles motivate the multimodal architectures discussed in Section 3.3.4. No single sensing domain is suitable for every wildfire-monitoring task. Satellite and aerial systems are most suitable for regional surveillance, hotspot monitoring, large-scale fire-risk estimation, and fire-spread monitoring because they provide broad and repeatable spatial coverage. However, their usefulness for very early ignition detection is limited by revisit intervals, atmospheric conditions, cloud and smoke occlusion, mixed pixels, and reduced sensitivity to small fires [39,40,83].

UAV systems are most suitable for local smoke and flame detection, hotspot localization, tactical verification, and fire-front monitoring because they provide high spatial resolution and flexible visible, thermal, or multispectral imaging. However, their operational use is constrained by flight endurance, weather sensitivity, communication range, payload limitations, safe navigation requirements, and regulations [55,91,92,93]. IoT and WSN systems are most suitable for continuous local early warning in high-risk areas because they can measure local environmental variables at fine temporal resolution. They can identify abnormal temperature, humidity, gas, smoke, or wind patterns and can support low-latency alerts through edge or fog computing. Their main limitations are restricted spatial coverage, node-density requirements, energy use, connectivity failures, and maintenance needs [27,29,45,137]. Local and specialized systems provide continuous site-specific observation through fixed cameras, weather stations, air-quality monitors, and embedded acoustic devices. Their effectiveness depends on sensor placement, local infrastructure, calibration, and maintenance [18,124,125].

Overall, the reviewed evidence indicates that multimodal wildfire-monitoring architectures are needed because the sensing domains provide complementary capabilities. Satellite and fire-weather products can provide regional context; IoT and WSN systems can detect local environmental anomalies; and UAVs or fixed cameras can support targeted visual verification. This combination may improve coverage, timeliness, and situational awareness, but it also introduces challenges related to data synchronization, communication reliability, energy use, interoperability, system cost, and missing sensor streams [22,100,101,120,138].

In practical terms, IoT/WSN and fixed local sensors are most suitable for continuous local early-ignition warning in instrumented high-risk areas because they provide frequent in-situ measurements. UAVs and fixed cameras are most suitable for rapid visual smoke or flame detection, local hotspot localization, and post-alert verification. Satellite and aerial remote sensing are most suitable for regional surveillance, active-fire mapping, and fire-spread or risk modeling, rather than very small or short-duration ignition events (see Table 4). Real-time deployment is most feasible through lightweight edge inference on local cameras, IoT gateways, and UAV platforms, although this requires trade-offs among latency, energy, communication reliability, and model complexity.

Having outlined the four principal sensing domains, the following section examines how AI methods are applied within and across these domains to support detection, segmentation, and risk prediction (RQ2).

### 3.2. AI Methods Across Wildfire Sensing Domains (RQ2)

This section examines how artificial intelligence (AI) techniques are applied across wildfire detection domains, building on the sensing-based taxonomy introduced in Section 3.1. To address Research Question 2 (RQ2), it focuses on how collected data are processed with machine learning and deep learning, and organizes the literature by the main learning tasks addressed by these models.

#### 3.2.1. Classical Machine Learning Approaches

Classical machine learning methods remain an important baseline in several wildfire monitoring settings, especially where data are structured, feature dimensions are moderate, or embedded deployment is required.

To provide a structured overview of the diverse AI approaches discussed across sensing domains, Table 5 summarizes the main model categories, their typical sensing contexts, and key observations. The categories span classical machine learning for risk prediction, deep learning for detection and segmentation, and recent edge and distributed learning approaches. A detailed mapping of individual studies, datasets, and reported metrics is provided in Appendix A (Table A1).

#### 3.2.2. AI Task Taxonomy

To address RQ2, we organize AI-enabled wildfire studies by task type rather than by sensing domain or algorithm family. Drawing on recent reviews and domain-specific studies across satellite, UAV, IoT/WSN, and local camera systems, we identify five main task families: (a) binary or multiclass fire detection; (b) smoke and early-stage detection; (c) segmentation and burned-area mapping; (d) risk prediction and forecasting; and (e) multimodal fusion and hybrid architectures [23,24,139].

In the detection family, the goal is to decide whether an image region or sensor window shows fire-related activity. Typical inputs are RGB or infrared images from satellites, UAVs, or fixed cameras, and in some cases environmental time series from IoT nodes. Classical machine learning models (e.g., SVMs and random forests) and convolutional neural networks, including lightweight and YOLO-style detectors, classify fire vs. non-fire or locate fire objects in scenes [59,140,141]. A closely related group focuses on smoke and early-stage detection, targeting thin, low-contrast plumes or subtle anomalies before flames are visible, often using specialized small-target detectors, attention mechanisms, or feature-entropy-guided networks to boost sensitivity in complex backgrounds and illumination conditions [142,143,144,145].

Segmentation and burned area mapping treat wildfire as a pixel-wise labeling problem, where encoder–decoder architectures such as U-Net variants, attention-based segmenters, and transformer-enhanced models delineate active fire regions, smoke masks, or post-fire scars in satellite, UAV, or CCTV imagery [20,67,146]. In the satellite domain, related work also uses deep generative and unsupervised methods on multi-temporal imagery to detect changes and burned areas without dense manual annotation [26,50]. A second family focuses on spatiotemporal prediction and risk forecasting, using time series of meteorological variables, fuel and land-cover descriptors, historical fire records, or remote-sensing indices to predict ignition or burn probability, spread, or severity with models ranging from regression and tree ensembles to CNN–LSTM hybrids, transformers, and hybrid physics–ML frameworks [147,148,149,150,151]. Some of these studies explicitly represent spatial dependence and multiscale drivers, while others produce daily or sub-daily risk indices for operational decision support [6,19]. A smaller but growing line of work addresses multimodal fusion and hybrid architectures, combining satellite imagery with weather or fire danger indices, UAV video with thermal and contextual information, and IoT-based streams that merge environmental sensors, camera feeds, and social data [22,64,86,122], together with federated, edge, and robustness-oriented training schemes that link model design to communication, energy, and privacy constraints in distributed wildfire monitoring networks [28,71,152].

Table A1 summarizes the AI landscape for wildfire sensing by grouping representative models within each task family and sensing domain, together with their data sources, deployment profiles, and reported metrics. The following Section 3.2.1, Section 3.2.2, Section 3.2.3, Section 3.2.4, Section 3.2.5 and Section 3.2.6 revisit these groups in more detail, examining classical machine learning methods, convolutional and encoder–decoder networks, object detection and transformer-based models, temporal and forecasting architectures, and edge or federated deployments.

Representative examples of these models and their reported metrics are summarized in Table A1 under the “Classical ML” task family. Reviews of machine learning applications in wildfire science report extensive use of algorithms such as logistic regression, support vector machines (SVMs), k-nearest neighbors, and tree-based ensembles as early tools for fire detection and risk estimation, often operating on tabular feature sets derived from remote sensing or environmental variables [139,153]. In satellite and remote sensing applications, these methods are typically trained on spectral indices, texture descriptors, or other handcrafted features to perform binary or multiclass classification of burned vs. unburned areas, fire vs. non-fire pixels, or different fire severity levels, and they continue to serve as benchmarks against which deep learning models are compared [6,148].

In IoT and WSN ground sensing networks, classical ML remains the dominant paradigm for anomaly-based early detection and local risk prediction. Studies of sensor-assisted wildfire detection and smart WSN architectures report the use of decision trees, random forests, gradient boosting machines, and other ensemble techniques trained on temperature, humidity, gas concentration, and wind measurements to distinguish normal conditions from fire suspect patterns [16,47,154]. These works often highlight the robustness of tree-based models to noise and missing data, as well as their relatively low computational cost and interpretability, which are important for battery-powered nodes and edge deployments [29,155]. Similar approaches are used in multi-sensor prediction systems that integrate environmental measurements with additional contextual variables, in which classical ML methods provide baseline forecasts of ignition probability or short-term fire occurrence [64,123].

Within local and specialized systems, including CCTV-based monitoring and social IoT frameworks, classical ML methods frequently act as lightweight filters or frontline detectors. For example, some camera-based systems extract color, motion, or texture features from video frames and pass them to SVM or random forest classifiers to flag candidate fire regions before more computationally intensive deep networks are invoked [156,157]. In IoT-centric architectures, rule-based logic is often combined with simple classifiers to trigger alerts or to prioritize which data streams should be analyzed by downstream deep learning models or sent to the cloud [27,122]. Across these domains, the literature suggests that classical ML is most competitive when datasets are modest in size, features are well engineered, and transparency, stability, and ease of deployment are prioritized over peak accuracy [139,153]. However, comparative studies generally find that convolutional and encoder–decoder networks outperform classical baselines on high-dimensional image and segmentation tasks when large labeled datasets are available, leading many recent systems to adopt hybrid designs where classical ML provides fast, interpretable screening and risk scoring, while deep models handle high-fidelity analysis on selected spatial or temporal subsets [24,86].

#### 3.2.3. Convolutional and Encoder–Decoder Networks

Convolutional neural networks (CNNs) now form the core of most image-based wildfire detection and segmentation systems, particularly in the binary/multiclass detection and segmentation/burned-area mapping task families. Representative CNN classifier and encoder–decoder segmentation models, together with their datasets and metrics, are summarized in Table A1 under the “CNN classifiers” and “Encoder–decoder CNNs” groups. Reviews of deep learning methods for forest fire surveillance emphasize that CNN-based pipelines have largely replaced handcrafted feature approaches for recognizing fire and smoke in satellite, UAV, and CCTV imagery, due to their ability to learn hierarchical spatial features under diverse backgrounds and illumination conditions [24,158]. In many studies, CNNs are adapted via transfer learning from ImageNet-pretrained backbones such as ResNet, EfficientNet, or YOLO variants, and then fine-tuned on wildfire datasets, including open-flame and smoke benchmarks and domain-specific collections [23,72,135].

A substantial subset of work uses encoder–decoder CNNs for pixel-wise segmentation of active fire regions, smoke masks, and burned areas, especially in satellite and UAV domains. U-Net and its derivatives remain common choices, often enhanced with lightweight modules or attention blocks to balance accuracy and computational cost [20,146]. For example, Zhang et al. [67] propose a lightweight segmentation network (LSNet) with parallel feature multiplication and attentional fusion for real-time wildfire detection, reporting competitive accuracy while maintaining low latency. Similar encoder–decoder designs are used for smoke segmentation in CCTV and aerial imagery, where optimized U-Net variants improve boundary delineation and sensitivity to small objects compared with plain encoder–decoder baselines [53,159].

Recent work pushes beyond standard architectures by incorporating multiscale context, attention mechanisms, and modern backbones. Studies such as those by Zhang et al. [59], Cao et al. [160], and Cheng et al. [149] show that feature pyramid structures, asymmetric fusion, and attention modules can enhance detection of heterogeneous fire signatures, including small flames and diffuse smoke. Transformer-augmented designs also appear in the literature: Choi et al. [51], Ghali et al. [52] and Yuan et al. [161] integrate Swin Transformer or window transformer blocks with CNN backbones to improve multiscale feature representation in UAV and ground camera imagery, leading to gains in detection robustness under cluttered scenes. For hyperspectral and satellite applications, CNNs are combined with temporal or spectral attention to better exploit rich spectral–temporal information [54,162].

At the same time, many systems focus on lightweight and deployable CNNs to support real-time inference on edge devices, UAVs, and low-power platforms. Work on pruned or compact networks, such as Pan et al. [163], Zhang et al. [59], and Yun et al. [164], demonstrates that carefully designed architectures can achieve high detection accuracy with reduced parameter counts and computation, making them suitable for embedded GPUs or microcontrollers. Camera-based systems using tailored CNNs or EfficientNet variants report detection accuracies above 90–95% on curated wildfire datasets, but also note that performance can degrade under domain shifts such as new camera viewpoints, vegetation types, or weather conditions [141,156]. Overall, the evidence indicates that convolutional and encoder–decoder networks provide a strong backbone for most image-centric wildfire tasks, while their robustness depends on dataset diversity, careful loss design, and, in some cases, integration with temporal or contextual modules discussed in later sections [24,139].

#### 3.2.4. Object Detection and Small Target Models

Object detection networks are now central to wildfire fire/smoke localization tasks, especially when real-time performance and small target sensitivity are required. As summarized in Table A1 under the “Object detection and small target models” group, recent work is dominated by YOLO family one-stage detectors for UAV and CCTV imagery, together with specialized small target and transformer-enhanced detectors. A large portion of recent work builds on the YOLO family, adapting versions such as YOLOv5, YOLOv7, YOLOv8, and more recent variants to detect flames and smoke in UAV, CCTV, and satellite imagery. Studies including Casas et al. [165], Yun et al. [164], Yun et al. [96], Shi et al. [145] and Zhang et al. [84] report high precision and recall on curated datasets, often after tailoring anchors, feature pyramids and loss functions to the specific characteristics of wildfire scenes. Specialized models such as Fire YOLO for small-target inspection and FFYOLO for lightweight detection in constrained environments further illustrate how YOLO backbones can be optimized for small, moving, or partially occluded fire objects [143,164].

Several works explicitly address small-target and smoke detection by modifying standard detectors or introducing new architectural components. Zhan et al. [142] propose PDAM–STPNNet for small-target smoke detection in satellite imagery, combining attention mechanisms and spatio-temporal modules to enhance weak signals. Guan et al. [144] introduce a feature entropy-guided neural network to improve discrimination of subtle fire features from cluttered backgrounds, while Fire YOLO and related methods adjust receptive fields and multiscale feature fusion to better capture small fires and thin smoke plumes [143,145]. On UAV imagery, Mukhiddinov et al. [55] and Saydirasulovich et al. [93] show that optimized YOLOv5 and YOLOv8 variants can detect smoke and fire at high frame rates, making them suitable for onboard or near real-time surveillance.

Beyond one-stage detectors, two-stage and transformer-based models are increasingly explored for robust wildfire detection under complex backgrounds. Faster RCNN remains a common baseline in comparative studies, particularly for high-resolution satellite and CCTV data, although its higher computational cost can limit real-time deployment [165,166]. Transformer-augmented detectors aim to improve multiscale representation and contextual reasoning: Ghali et al. [52] and Muksimova et al. [167] integrate transformer blocks with CNN backbones for UAV-based fire detection and segmentation, while Choi et al. [51] propose an Optimized Faster R CNN with Swin Transformer to enhance robustness for multiclass wildfire detection. In parallel, models such as MCDet and FF Mamba YOLO explore RGB–thermal fusion and state space mechanisms to further boost performance on challenging small target scenarios [43,168]. Collectively, these studies indicate that YOLO style one stage detectors currently dominate operational wildfire localization, with transformer-enhanced and specialized small target architectures offering promising improvements where computational budgets allow.

#### 3.2.5. Temporal and Forecasting Models

Temporal and forecasting models treat wildfire as a spatiotemporal prediction problem, rather than a purely static detection task. Table A1 summarizes representative spatio-temporal and forecasting approaches under the “Spatio-temporal ML—occurrence and spread forecasting” and “Multi-source & multi-type fusion” task families, including their data sources, deployment profiles, and reported metrics. In this family, models ingest time series of meteorological variables, fuel and land-cover descriptors, fire history, and remote-sensing indices to estimate ignition probability, burn probability, spread rate, or future fire extent. Reviews of machine learning applications in wildfire science highlight that early work relied on regression, decision trees, and ensemble methods, but that recent studies increasingly adopt deep architectures capable of capturing nonlinear temporal dependencies and spatial context [139,153]. Examples include next-day or daily risk predictors that integrate weather and vegetation conditions, as well as models that explicitly represent spatial patterns of drivers across landscapes [6,148].

Several studies develop time series and spatiotemporal models that operate on gridded or station-based data. Farguell et al. [147] propose a machine learning framework to estimate fire arrival time from satellite active fire products, effectively forecasting front propagation given previous detections. Zhai et al. [169] and Gholami et al. [170] explore learning-based wildfire spread and risk prediction, using historical fire records and environmental covariates to model how fires evolve in space and time. More recent work introduces clustering and spatial scale analysis to capture regional heterogeneity in fire drivers, allowing models to adapt to differing climatic and fuel regimes across a study area [6,19]. These approaches typically use gradient boosting machines, random forests, or deep feed-forward networks, sometimes augmented with lagged variables and autoregressive components [151].

Deep learning is increasingly used for multimodal and sequence-based forecasting. Xu et al. [66] built a spatiotemporal wildfire prediction system that fuses multimodal data, including meteorological fields and remote sensing indicators, within a deep architecture designed to learn both spatial and temporal dependencies. Cheng et al. [149] propose a deep learning-based prediction model that leverages neural networks for regional wildfire risk estimation, while Deng et al. [150] focus on daily wildfire risk prediction using machine learning pipelines trained on operational weather and fire datasets. At the same time, multi-source fusion studies, such as those by Bera et al. [123], Muhaimin et al. [64], and Zhu et al. [22], combine sensor networks, satellite observations, and environmental variables within recursive transformer or multimodal fusion frameworks to enable very early detection horizons and short-term forecasting.

A smaller but growing body of work examines robustness, federated training, and hybrid physics–ML schemes within temporal models. Ide and Yang [152] investigate adversarial robustness for deep wildfire prediction models, showing that small input perturbations can significantly affect risk estimates and motivating more resilient training strategies. Federated approaches proposed by Supriya and Gadekallu [71] and Venkateswaran et al. [171] distribute training across clients or regions without centralizing raw data, which is particularly relevant for privacy-sensitive or bandwidth-limited fire risk applications. Hybrid methods that combine physical insights with data-driven components, such as those explored by Ji et al. [62] and Bera et al. [123], demonstrate that incorporating domain knowledge about fire behavior and fuels can improve generalization across regions and fire regimes. Taken together, these studies show that temporal and forecasting models are becoming an integral part of AI-enabled wildfire systems, providing forward-looking risk information that complements the instantaneous detection capabilities of CNN and detector-based pipelines.

#### 3.2.6. Edge, Embedded and Federated Learning

A growing subset of the literature focuses on how AI models are deployed in resource-constrained and distributed environments, including UAVs, IoT/WSN nodes, and edge gateways. As summarized in Table A1 under the “Edge/embedded CNNs—low power deployment” and “Federated learning—distributed detection and prediction” task families, these studies explicitly characterize models by their deployment profile, latency, and resource requirements, in addition to accuracy metrics. In these settings, the main challenges are latency, energy consumption, memory footprint, and communication cost, rather than only peak accuracy. Studies on IoT- and WSN-based wildfire detection show that lightweight classifiers and carefully designed architectures can run directly on sensor nodes or fog/edge layers, enabling rapid local decision-making and reducing the need to stream raw data to the cloud [27,28,29]. Similar trends are observed in social IoT and multimodal frameworks, where embedded AI components perform initial filtering or risk scoring before forwarding alerts or high-value data for more computationally intensive analysis [122,155].

On the vision side, several works design or optimize CNN and detector architectures specifically for deployment on drones, embedded GPUs, and low-power devices. Pruned and compact networks demonstrate that it is possible to maintain strong detection performance while significantly reducing parameter counts and inference time, which is critical for real-time aerial surveillance [59,163]. Edge-oriented systems such as those of Peruzzi et al. [48] and Üremek et al. [28] combine image and audio processing on low-power hardware, highlighting trade-offs between model complexity, detection accuracy, and battery life. In UAV-based monitoring, lightweight YOLO variants and customized CNNs are frequently chosen to meet onboard compute constraints while delivering flame or smoke detection at frame rates [55,96].

Federated and distributed learning have recently emerged as complementary strategies for training wildfire models without centralizing all data. Supriya and Gadekallu [71] propose a particle swarm-optimized federated learning framework for early forest fire detection, showing that distributed clients can collaboratively train a model while keeping raw sensor data local. Venkateswaran et al. [171] extend this idea to wildfire area prediction, illustrating how federated training can exploit geographically dispersed datasets while addressing privacy and bandwidth constraints. Robustness-oriented work by Ide and Yang [152] further underscores the need to account for adversarial perturbations and distribution shifts when deploying deep models in operational wildfire systems, a point that is particularly relevant for federated and edge settings where data quality and distributions may vary across clients.

In the context of this review, these studies suggest that deployment considerations are now integral to AI model design for wildfire monitoring. Edge and embedded systems favor tiny or pruned networks, quantization and on-device inference; IoT and WSN architectures rely on energy-aware protocols and simple but robust models at the node or fog layer; and federated frameworks enable collaborative training across regions while respecting data governance constraints [27,71,163], as reflected in the deployment profiles reported for these models in Table A1.

The reported performance measures should be interpreted within their task and dataset context. Classification studies commonly report accuracy, precision, recall, or F1-score; object-detection studies report mAP; segmentation studies report IoU or Dice scores; and risk or spread prediction studies report RMSE, MAE, or $R^{2}$. These measures are not directly comparable across tasks. Therefore, this review does not identify one best AI model for all wildfire-monitoring applications. Instead, lightweight detection models are most relevant to real-time camera and UAV monitoring, segmentation models are useful when fire boundaries are needed, and spatio-temporal models are useful for risk and spread forecasting.

Having reviewed how AI methods are applied within individual sensing domains, we next turn to the broader challenges that arise when these methods must generalize across regions, integrate across modalities, and operate reliably under real-world uncertainty (RQ3).

### 3.3. Cross-Domain Challenges: Generalization, Integration, and Uncertainty (RQ3)

**Figure 4:**
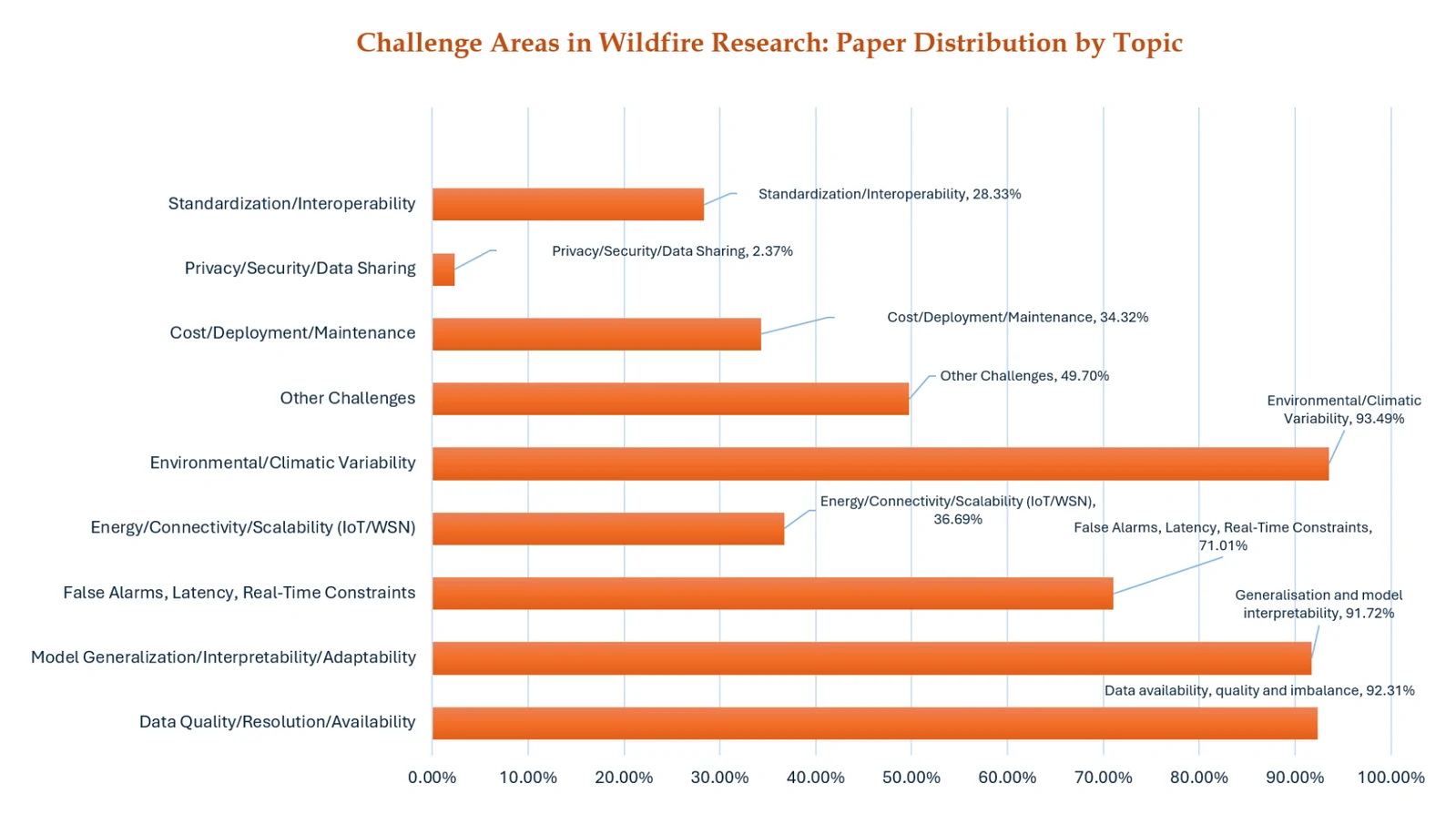
Frequency of technical and operational challenges reported across the 169 studies included in the PRISMA-guided synthesis. Challenge categories were non-exclusive; therefore, one study could be coded in more than one category. Bar labels show the number of coded studies and the percentage calculated.

Wildfire detection and early warning systems face a recurring set of technical and operational challenges that cut across sensing domains, modeling approaches, and deployment contexts. To understand which issues are most frequently highlighted in recent work, we manually coded each included paper against a predefined set of challenge categories. To construct the challenge taxonomy in Figure 4, the two reviewers who conducted full-text screening independently coded each of the 169 included studies against nine predefined challenge categories (data availability/quality/imbalance; generalization and interpretability; false alarms/latency/real-time constraints; energy, connectivity, and scalability; environmental/climatic variability; cost, deployment, and maintenance; privacy, security, and data-sharing; standardization and interoperability; and other challenges, including annotation scarcity and small-target detection). A paper was coded as addressing a category if it explicitly discussed that challenge in its results, discussion, or limitations sections. Coding disagreements were resolved through discussion, consistent with the screening procedure described in Section 2. Because papers frequently addressed multiple categories, the percentages in Figure 4 are not mutually exclusive and do not sum to 100%. Figure 4 summarizes the resulting distribution.

Figure 4 shows that environmental and climatic variability (93.49%), data quality, resolution, and availability (92.31%), and model generalization, interpretability, and adaptability (91.72%) were the most frequently reported challenges. False alarms, latency, and real-time constraints were also common (71.01%). These issues are closely linked because models are often developed using datasets from limited regions, sensors, seasons, and fire regimes. Such limitations can reduce transferability and increase false alarms when systems are applied in new environments.

In contrast, the reviewed literature contains fewer clear examples of studies that evaluate solutions designed to address these problems, such as multi-region benchmark datasets, spatial or temporal holdout testing, external validation, domain adaptation, uncertainty-aware prediction, and federated learning. Federated learning can support distributed wildfire-sensing networks by allowing models to be trained across separate sites without centralizing all local data, but it remains an emerging approach in the reviewed literature. This difference suggests that the field identifies important barriers to operational deployment more often than it provides validated solutions for overcoming them.

#### 3.3.1. Data and Generalization Gaps

As Figure 4 indicates, the majority of recent studies explicitly acknowledge issues related to data availability, quality, and imbalance (92.31%), and a substantial proportion raise concerns about model generalization and interpretability (91.72%), underscoring that these are pervasive, cross-cutting challenges rather than isolated problems.

Table 6 shows that the literature more frequently reports internal detection or prediction performance than rigorous evidence of operational transferability. Random within-dataset splits are common in local image, sensor, and fire-risk datasets, whereas spatial holdouts, temporal holdouts, cross-region tests, cross-sensor tests, and independent external validation remain limited across most sensing domains. Stronger examples include Sen2Fire, which uses geographically separate training, validation, and test areas, and EO4WildFires, which includes multi-sensor wildfire data from 45 countries [58,135]. Similarly, real-time deployment is more commonly evaluated in UAV, IoT/WSN, and local embedded systems than in satellite-based workflows, whereas formal uncertainty quantification is mainly found in a small number of cross-cutting forecasting and robustness studies. These gaps indicate that high performance on local datasets should not be interpreted as evidence of reliable deployment across new regions, fire regimes, sensor configurations, or environmental conditions.

Jain et al. [139] emphasize that although machine learning models for wildfire detection and prediction often report strong accuracy on their development datasets, they are rarely stress-tested under substantial spatial or temporal shifts, which limits their real-world reliability. Deep learning surveys for wildland fire remote sensing similarly conclude that many CNN and transformer models exceed 90% accuracy, F1, or IoU on curated image collections, but that these results are typically obtained on single-region, single-sensor benchmarks with homogeneous conditions (e.g., specific forest types or national fire archives). This problem is important because wildfire conditions can change across regions and over time.

New benchmark datasets aim to improve cross-region and multi-sensor evaluation. Sykas et al. [58] introduce EO4WildFires, which combines Sentinel-2, Sentinel-1 SAR, and meteorological data for more than 31 000 wildfire events across 45 countries. Xu, Berg, and Haglund [135] introduce Sen2Fire, which uses geographically separate training, validation, and test areas for satellite fire detection. These datasets provide stronger evaluation settings, but model performance remains sensitive to data quality, event distribution, sensor selection, and atmospheric conditions.

However, most fire-risk and spread-prediction models are still trained and tested using data from one region. Many studies use random data splits from the same geographic area and do not test performance across regions, seasons, fire regimes, or sensor systems [139]. Therefore, it remains difficult to assess whether these models can perform reliably under new fire conditions or climate extremes.

A second gap is the absence of standardized cross-domain benchmarks that jointly cover the main sensing modalities discussed in Section 3.1, namely satellite and aerial imagery, UAV data, IoT/WSN ground measurements, and local camera or other specialized sensor streams. Many multimodal studies use custom datasets from one study area. This limits direct model comparison and reduces evidence of transferability across sensor combinations and ecosystems [22,58,139].

Addressing these data and generalization gaps will require coordinated efforts to: (i) design multi-region, multisensor benchmarks with held out geographic and temporal test sets (e.g., EO4WildFires and Sen2Fire style datasets extended to additional domains); (ii) report explicit cross region and cross sensor transfer experiments as part of model evaluation; and (iii) integrate physically meaningful features and domain knowledge, such as fire weather indices, fuel models, and topographic controls, to reduce overfitting to local training conditions [22,58,139].

#### 3.3.2. Imbalance, Annotation Scarcity and Early Stage Detection

Barmpoutis et al. [13] review early forest fire and smoke detection systems and note that camera-based datasets are often imbalanced. They contain many negative images or images of clear flames, but few examples of early smoke. This can reduce model performance when smoke is faint, small, or partly hidden. Early smoke also occupies few pixels and can look similar to clouds, haze, or background features. Therefore, good results on small and curated datasets may overestimate real-world performance. Although these issues are captured under the broader “other challenges” slice in Figure 4, their practical impact is substantial because they directly constrain early-stage smoke and small target detection.

Di Martino et al. [26] show that modern detectors still struggle with small and low-contrast smoke plumes. Limited labeled early-smoke data often leads researchers to use data augmentation and synthetic samples. These methods can improve detection results, but models may still produce false alarms in complex backgrounds or miss weak smoke at long distances. Recent small-target and lightweight YOLO models use multiscale features, attention modules, specialized detection heads, and pruning to improve detection of small fire and smoke targets while supporting edge deployment [128,142,163]. However, many studies use custom datasets with limited early-ignition diversity. More real and well-labeled early smoke and ignition data are needed to test whether these models work across different landscapes, weather conditions, sensors, and fire regimes.

Lightweight YOLO and other small-target detection models use multiscale features, attention mechanisms, specialized detection heads, transfer learning, and pruning to improve detection of small fire and smoke targets while supporting edge deployment [128,142,163]. These methods can improve detection accuracy and reduce computing requirements. However, many studies use custom datasets with few early-ignition samples. Therefore, their performance may not transfer well to different landscapes, weather conditions, camera settings, sensors, or fire regimes. Larger and better-balanced datasets with real early-smoke and ignition cases are still needed.

These limitations are especially important in remote and data-scarce regions. Such regions may have few labeled early-ignition images, limited smoke annotations, and little local data for camera or sensor calibration. They may also have limited power supply, communication coverage, maintenance access, and computing resources. These constraints can delay detection or prevent reliable model deployment when a fire is still small. In operational wildfire management, early-warning systems in data-scarce regions should therefore be designed as staged decision-support workflows rather than as stand-alone classifiers. Lightweight local models can generate an initial alert from camera, UAV, or sensor data, but the alert should be transmitted through reliable low-bandwidth communication where possible, assigned a confidence level, and rapidly verified using an available complementary source, such as a fixed camera, patrol, UAV, satellite observation, or local incident-reporting channel. Local calibration data remain important for reducing false alarms caused by region-specific vegetation, terrain, weather, sensor placement, and background smoke or haze. Where communication, energy, or maintenance capacity is limited, systems should prioritize robust edge inference, store-and-forward communication, sensor-health monitoring, and clearly defined escalation procedures for fire-management personnel.

#### 3.3.3. Deployment, Uncertainty and Robustness

Supriya and Gadekallu [71] show that cloud-based wildfire prediction can face communication and latency problems in remote areas with limited bandwidth. Their federated learning approach trains models locally and shares model updates instead of raw data. This can reduce communication costs and support distributed learning. However, deployment-ready AI must still consider bandwidth, privacy, and on-device computing requirements.

Consistent with Figure 4, energy, connectivity, and scalability challenges were reported in 62 out of 169 studies (34.4%), while privacy, security, and data-sharing issues were reported in only 4 studies (2.2%). This finding suggests that privacy and data-governance considerations remain comparatively under-examined in the reviewed wildfire-monitoring literature.

Edge AI is important for continuous monitoring on IoT nodes, UAVs, and local camera systems. Lightweight CNN and YOLO models can reduce latency by processing data near the sensor instead of sending all data to the cloud. Üremek et al. [28] show that edge systems need to balance model accuracy with energy use, memory limits, and response time. However, battery life, unstable connectivity, harsh weather, and hardware failures can reduce reliability during long monitoring periods.

Supriya and Gadekallu [71] further emphasize that federated settings introduce their own robustness challenges, including heterogeneous local data distributions, client dropout, and the risk of model poisoning attacks. Venkateswaran et al. [171] extend that models trained on historical weather, fuel, and fire data may also perform poorly under new climate conditions or changing ignition patterns. Therefore, robustness should be tested under realistic deployment conditions, including limited connectivity, missing data, changing environmental conditions, and partial system failure.

Uncertainty quantification remains limited in many wildfire AI studies. Most detection and prediction models provide one output without showing how confident the model is. Ide and Yang [152] show that uncertainty-aware fire-danger models can provide both risk estimates and uncertainty information. This can help decision-makers identify high-risk areas with stronger confidence and avoid over-reliance on low-confidence predictions. However, uncertainty analysis is still uncommon in edge-camera, UAV, and satellite detection systems.

Overall, deployment-ready wildfire AI should be evaluated across the edge–federated–cloud system. Evaluation should include accuracy, latency, energy use, communication cost, privacy, reliability, and uncertainty. Systems should also be tested under sensor failure, missing data, unstable networks, and changing environmental conditions. These tests are needed to support reliable decision-making in real wildfire operations.

#### 3.3.4. Integration and Multimodal Fusion Challenges

Standardization and interoperability issues are mentioned in only 30.18% of the reviewed studies (Figure 4), reflecting limited awareness or attention to how ad hoc, project-specific designs make it difficult to combine data streams or compare systems across regions.

Table 7 shows that no single fusion strategy is suitable for all wildfire-monitoring settings. Early and intermediate fusion can use more detailed information when synchronized data and sufficient computing resources are available. Decision-level fusion and system-level orchestration may be more suitable for distributed systems because they can combine sensors with different sampling rates and may continue working when one sensor stream is unavailable. However, few studies directly compare fusion strategies, test missing sensor streams, or report comparable latency and edge-hardware requirements. Therefore, the practical advantages of each fusion strategy need further testing in field deployments.

Zhu et al. [22] show that combining multiscale UAV imagery with other data sources can significantly improve smoke and fire detection in complex terrain, but they also stress that most fusion pipelines are custom-built for a single study area and sensor setup. Their multiscale UAV framework uses attention and feature-fusion blocks to better capture small smoke plumes in cluttered scenes, yet the integration of satellite and ground data remains ad hoc and is not evaluated as a reusable, end-to-end multimodal pipeline.

Xu et al. [66] present a spatio-temporal wildfire-prediction framework that integrates multimodal time-series data. The study illustrates the potential value of combining environmental and remote-sensing information for wildfire-risk prediction. However, performance comparisons remain difficult because multimodal studies use different tasks, datasets, sensing configurations, and validation protocols. Bhamra et al. [138] introduce SmokeyNet, a deep network that fuses satellite fire detections, meteorological sensor data, and optical camera images for multimodal smoke detection, achieving higher accuracy and faster warnings than single-source baselines. At the same time, they acknowledge that reliance on multiple asynchronous data sources complicates data fusion, increases processing complexity, and may introduce latency in real-time deployments, particularly when any modality becomes unavailable or degraded.

Muhaimin et al. [64] combine visual fire classification with meteorological sensor-based risk prediction through weighted decision-level fusion. Their system integrates YOLOv11, ViT–GRU, and LSTM–Optuna models. This approach shows how visual detections can be combined with independent risk estimates, although its study-specific dataset and task design limit direct comparison and transferability assessment. Zhang et al. [172] and related work on forest fire segmentation highlight similar patterns: deep architectures with repeated deep–shallow feature fusion improve segmentation quality in UAV imagery, yet they typically focus on one or two modalities (e.g., RGB UAV video plus basic meteorology) and do not generalize to the full satellite–UAV–IoT stack summarized in Table A1.

Bera et al. [123] and related studies integrate multi-sensor environmental datasets for early fire-occurrence prediction. These studies show the value of combining environmental and geospatial variables, although the inputs, tasks, and fusion mechanisms vary substantially across studies. Across these works, multimodal fusion strategies vary widely, including early feature-level concatenation, attention-based cross-modal transformers, and late decision-level fusion, but they are almost always tailored to a single dataset, with different pre-processing pipelines, temporal resolutions, and output tasks. This heterogeneity mirrors the patterns seen in Section 3.2.1, Section 3.2.2, Section 3.2.3, Section 3.2.4, Section 3.2.5 and Section 3.2.6: while fusion is increasingly used in detection, segmentation, and forecasting models, there are still no standardized end-to-end multimodal benchmarks that cover satellite, UAV, IoT/WSN, and local camera streams together, and few studies report systematic ablation or cross-domain transfer experiments for their fusion strategies.

Taken together, the challenges reviewed in this section, spanning data and generalization gaps, annotation scarcity, deployment uncertainty, and multimodal integration, indicate that current AI-based wildfire systems remain heavily reliant on physical and meteorological sensing streams, with comparatively little attention paid to complementary, non-conventional sources of environmental information. This observation motivates the exploratory discussion that follows, in which we consider whether biological indicators, organisms that respond measurably to the same environmental drivers underpinning wildfire risk, might offer an additional, currently underexploited sensing layer for future hybrid early-warning frameworks.

## 4. Future Directions: Bioindicator-Based Hybrid Sensing

The previous sections presented a systematic review of AI-based wildfire sensing across four technical domains and examined the challenges these systems still face. This section shifts to an exploratory discussion, as outlined in Section 1. We examine whether biological signals could provide a complementary layer of information for current wildfire monitoring systems.

While existing wildfire detection systems primarily rely on engineered sensing infrastructure and AI-driven data analysis, recent research has begun exploring complementary sensing paradigms inspired by natural systems. In particular, biological organisms that respond sensitively to environmental changes may provide an additional layer of information to support early detection and risk assessment. This section examines such hybrid approaches, focusing on bioindicator-based sensing and its potential integration into conventional wildfire monitoring frameworks.

Pausas and Parr [32] argued that many animal species in fire-prone ecosystems have evolved traits and behaviors shaped by recurrent fire, which suggests that animals can serve as indicators of fire regimes and disturbance patterns. Engstrom [31] similarly reviewed first-order fire effects on animals and highlighted that fire can directly alter animal survival, movement, and habitat use. These studies support the broader idea that bioindicators are organisms whose responses reflect environmental conditions and can therefore be used as sentinels of environmental change. Building on this, Sanderfoot et al. [33] showed that wildfire smoke affects the health and behavior of wildlife across multiple taxa, while Albery et al. [34] linked wildfires to changes in wildlife disease patterns. At the community level, Lindenmayer et al. [173] found that bird occurrence is influenced by time since fire and changes in vegetation structure, and Johnson et al. [73] reported that Acorn Woodpecker movements and social networks shift in response to wildfire smoke. Mendyk et al. [174] provided evidence that the lizard Tiliqua rugosa shows an olfactory-driven response to smoke, which they interpreted as fire-avoidance behavior, further illustrating that animals can react strongly to fire cues. From a sensing perspective, Permana et al. [175] demonstrated that bird vocalizations can be classified using convolutional neural networks to distinguish normal calls from alarm calls, and proposed this as an early warning method for forest fires. More recent work by Ashok et al. [176] has explored integrated ecological surveillance systems that combine early fire prediction with biodiversity or species monitoring, reinforcing the potential to link hazard detection with ecological indicators. Taken together, these studies indicate that bioindicator species and their behavioral or acoustic signals provide a complementary, though still under-explored, pathway for sensing fire-related environmental stress, which motivates the conceptual exploration of bioindicator-based signals alongside conventional physical sensors in wildfire monitoring frameworks.

### 4.1. Bioindicators in Environmental Monitoring

Bioindicators are organisms whose physiology, behavior, or community composition reflects environmental conditions that are difficult to capture through physical measurements alone. In this review, we focus on honey bees as a well-studied example, building on evidence that links bee health, activity, and acoustic behavior to changes in temperature, humidity, air quality, and resource availability [74,177,178]. These studies show that bee colonies integrate exposure to multiple stressors over time, so that shifts in colony performance, foraging, or hive microclimate can act as indirect indicators of surrounding conditions.

**Figure 5:**
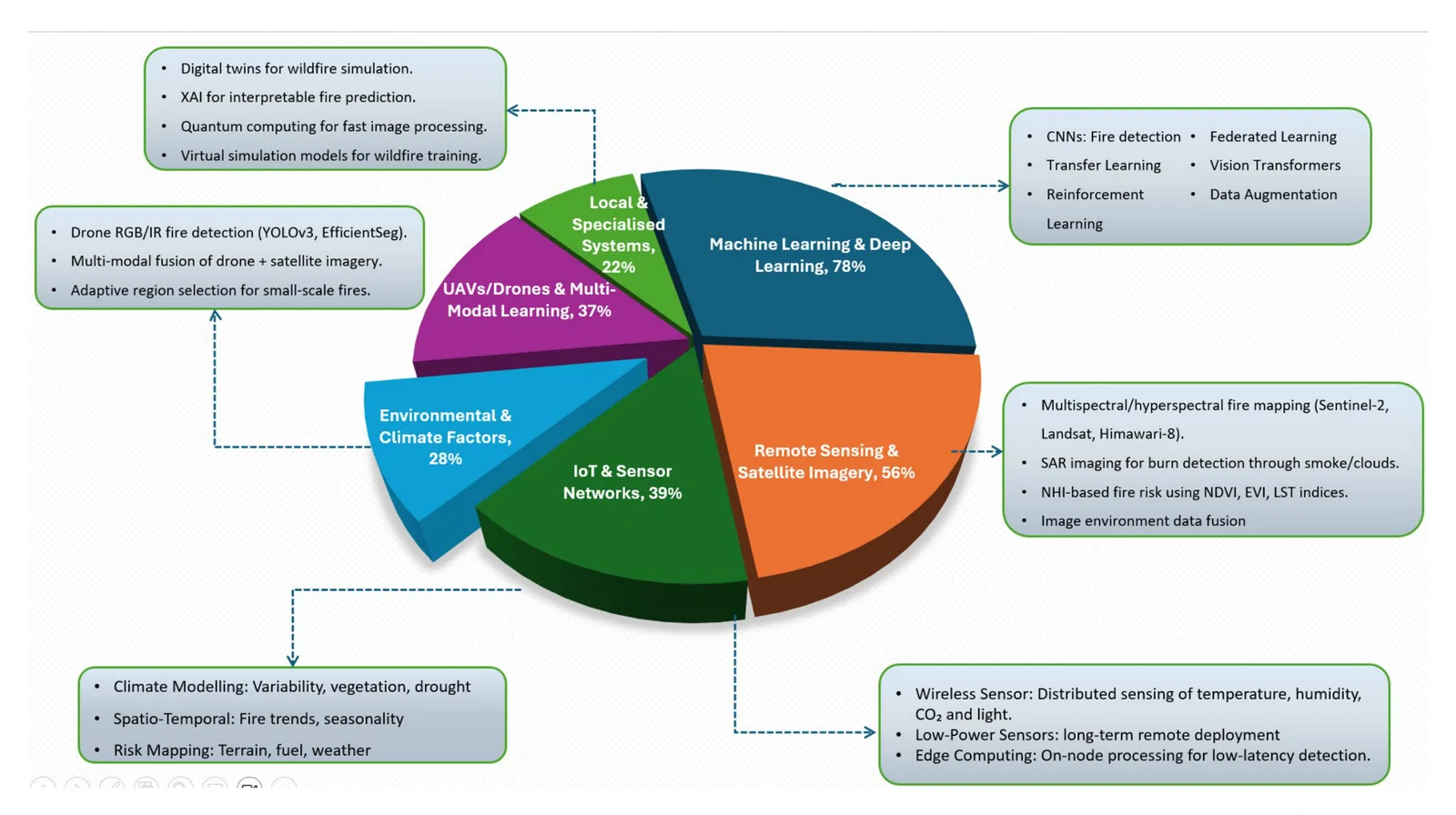
Thematic distribution of technological and environmental themes in the reviewed wildfire detection and early warning literature.

Wildfire activity is driven by a relatively small set of environmental factors, including temperature, humidity, fuel moisture, drought, wind, and vegetation state [1,129,139]. These same variables already appear across wildfire detection systems: satellite models use vegetation and smoke indices, IoT/WSN systems track temperature and humidity, and forecasting models combine weather and fuel metrics [10,45,111]. As shown in Figure 5, most of the reviewed literature is organized around these shared environmental and climate factors, alongside AI, remote sensing, and IoT/WSN themes, with many studies combining multiple modalities in hybrid frameworks.

Bioindicator studies show that pollinators respond strongly to these same drivers. Abou Shaara et al. [74,177] show that honey bees exhibit clear changes in survival, activity, and colony performance across temperature and humidity gradients, and that colonies actively regulate hive microclimate. Khan et al. [178] report that poor air quality alters bee foraging and colony health, similar to how smoke and drought affect fire-relevant vegetation and air quality. These findings suggest that pollinators experience many of the same stressors that underpin fire danger indices.

Together, this evidence positions bioindicators as an additional sensing layer on top of the environmental drivers already central to wildfire detection. Instrument-based systems measure temperature, humidity, and smoke directly, while bioindicator species integrate these same factors into behavioral and acoustic signals over time. This shared environmental foundation bridges Section 3.1, Section 3.2 and Section 3.3, which cover sensor and model-based monitoring, and the following subsections, which examine how bee acoustic behavior could serve as a nature-inspired indicator within hybrid early warning frameworks.

### 4.2. Bee Acoustics and Behavior Under Environmental Stressors

Honey bee colonies respond strongly to several environmental drivers that also underpin wildfire danger: temperature and humidity, air quality, and climate-driven changes in floral resources. These responses appear in foraging behavior, colony performance, microclimate regulation, and, in some cases, hive-level acoustic activity, offering a biological lens on the same conditions monitored by conventional wildfire sensors.

Temperature and humidity are primary drivers of bee stress. Abou Shaara et al. [74,177] show that survival, foraging, and thermoregulation in honey bees are highly sensitive to temperature and humidity, with colonies actively regulating hive conditions within narrow ranges. Heat wave studies further show that elevated temperatures alter colony behavior and physiology, from individual bees to whole-colony dynamics [179,180].

Air quality stressors produce similar effects. Bencsik et al. [76] show that in-hive CO_2_ levels reflect both environmental conditions and colony health, while Khan et al. [178] report that air pollution disrupts bee foraging, navigation, and colony health. These findings suggest that bees are sensitive to degraded air quality, similar to conditions produced by wildfire smoke.

Climate-driven changes in vegetation and forage also affect bee outcomes. Gounari et al. [181] show that weather variables affect hive productivity, while post-fire studies indicate that wildfire severity and landscape change can reshape bee diversity and plant–pollinator interactions [182,183]. These results show that the same climatic and vegetation shifts driving fire risk also affect bee foraging and colony outcomes.

Some of these stressors are directly reflected in hive acoustics. Bencsik et al. [184] show that specific vibrational and acoustic patterns correlate with colony state and external stress, while Kanelis et al. [78] show that features such as wing-beat frequency can be used with machine learning to decode colony behavior and stress status.

Overall, this evidence indicates that bee colonies act as integrated biological sensors of temperature, humidity, air quality, and resource conditions, the same factors that drive fire danger indices. This motivates the use of bee acoustic and behavioral signals as a complementary sensing layer that could, in principle, support early or corroborating indicators of wildfire-related environmental stress. These patterns are summarized in Table 8, which contrasts normal bee behavior with deviant responses under environmental and climatic stressors.

The table summarizes typical colony behavior under favorable conditions and the main behavioral or physiological deviations observed under heat, humidity, air quality, and resource-related stressors in the studies reviewed in Section 4.2. Environmental variables correspond to key drivers of wildfire risk, such as temperature, humidity, drought, and degraded air quality.

### 4.3. AI Models for Bee Acoustic and Environmental Monitoring

Building on Section 4.2, a growing set of studies applies machine learning and deep learning to bee sounds and hive sensor data to classify colony states and detect stress. A detailed overview of these applications, including tasks, inputs, and reported performance, is provided in Appendix B (Table A4).

Smart hive systems typically fuse hive temperature, humidity, and weight with acoustic or activity signals to detect anomalies and forecast colony health, with edge-based classifiers reporting real-time detection accuracies above 90% [201,202]. Acoustic-only approaches also perform well: deep learning models based on hive sound spectrograms classify colony states with 90–95% accuracy, and wing-beat frequency features can identify species and detect pre-swarm signals up to two days in advance [37,203]. TinyML-based systems extend this further by running lightweight audio classifiers directly on embedded hive devices, enabling low-bandwidth, real-time alerts [202].

Despite these promising results, the literature has important limitations. Most studies use small, controlled datasets with limited diversity in hive types and landscapes, and rarely test cross-site or cross-season generalization [204]. Critically, none of the studies in Table A4 link bee acoustic labels to actual wildfire or smoke events; stress labels instead come from colony inspections or pest records, which may not transfer well to wildfire-specific applications.

Overall, these AI applications show that bee acoustic and environmental signals can be reliably decoded into indicators of colony stress, using classification and forecasting approaches that closely parallel those used in wildfire detection. This methodological overlap suggests that architectures developed for wildfire sensor data could, in principle, be adapted for bee bioacoustic monitoring, providing a foundation for the hybrid architecture discussed next.

### 4.4. Conceptual Hybrid Architecture for Wildfire Monitoring with Bee Bioindicators

The previous sections showed that both wildfire systems and bee colonies respond to a common set of environmental drivers, including temperature, humidity, drought, and air quality [1,177]. Building on this shared foundation, we outline a conceptual hybrid architecture that treats bee colonies as an additional, nature-inspired sensing layer over the same environmental drivers.

**Figure 6:**
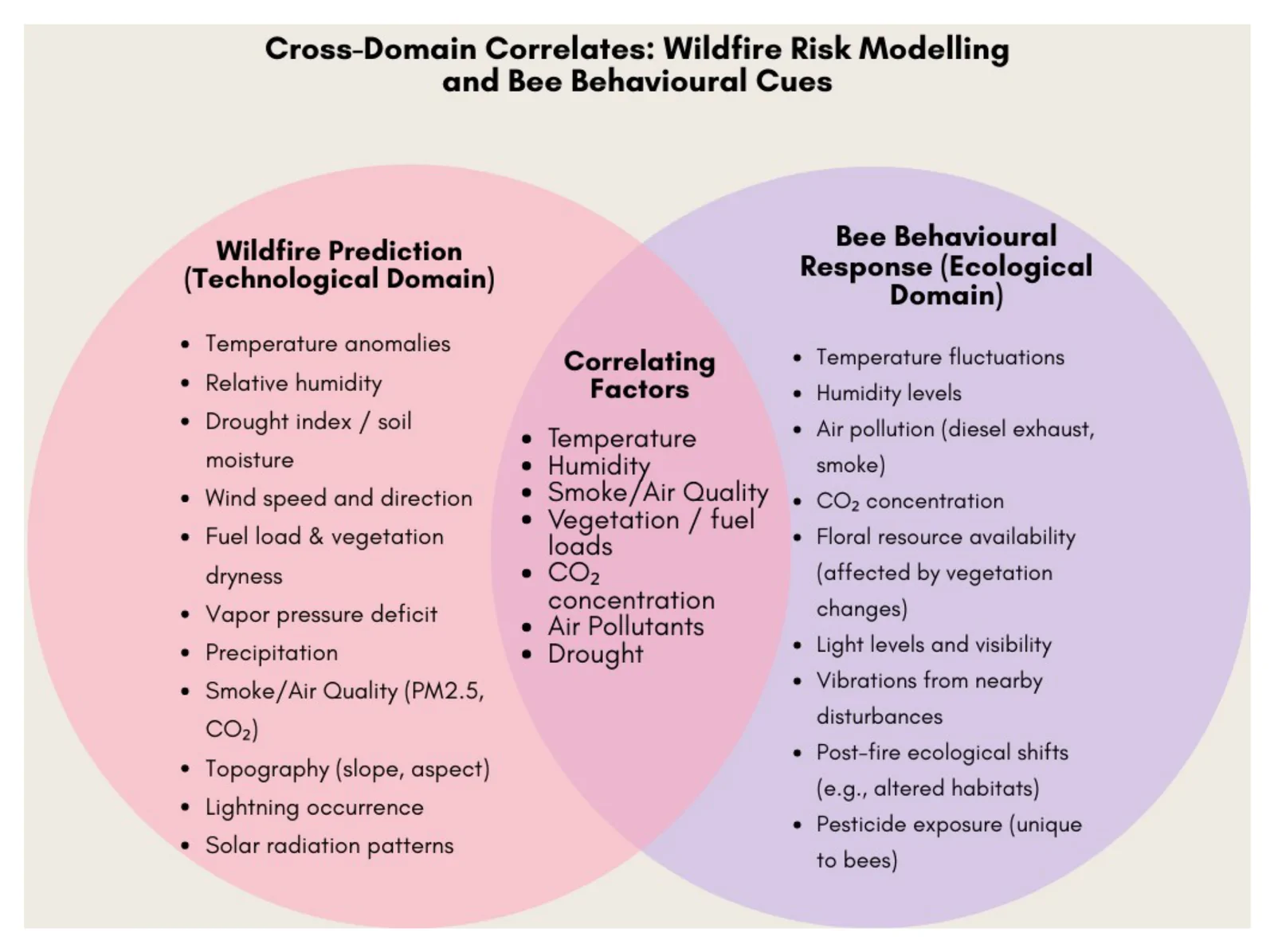
Cross-domain Venn diagram showing environmental factors that influence wildfire-risk models and bee behavioral responses. The overlap provides a conceptual basis for investigating whether bee acoustic and environmental signals could serve as a complementary contextual input in future hybrid wildfire-monitoring systems. This role has not yet been validated using wildfire-linked bee acoustic datasets.

This overlap is illustrated in Figure 6, a cross-domain Venn diagram showing shared and unique factors across wildfire prediction and bee behavior. Wildfire systems rely on variables such as temperature, humidity, drought indices, wind, and smoke products [1,139], while bee studies focus on temperature, humidity, air pollution, and floral resource availability, all of which influence foraging, thermoregulation, and hive acoustics [177,182].

Beyond these shared drivers, fire-ecology research reports that bee communities and species can respond to fire disturbance through mechanisms such as avoidance, resistance, and dormancy [38]. For example, some ground-nesting bees may survive fire by remaining deep in the soil, beyond the reach of lethal heat [38]. These findings demonstrate ecological sensitivity to fire-related disturbance, but they do not demonstrate that hive acoustic signals can detect wildfires, classify wildfire smoke, provide earlier warning than conventional sensors, or localize an ignition source.

As summarized in Appendix B Table A4, current bee-based studies support monitoring of colony states and environmental stress proxies, but do not validate direct wildfire detection, wildfire-smoke classification, earlier warning than conventional sensors, or ignition localization from bee acoustic signals. Hive responses are non-specific and may be influenced by colony condition, weather, management activity, wind direction, terrain, source distance, and smoke-plume transport; a hive coordinate identifies the hive location rather than the fire source. Bee-derived anomalies should therefore not operate as stand-alone wildfire alarms. Instead, they may be evaluated as low-confidence contextual features that prioritize verification only when georeferenced physical evidence, such as air-quality, meteorological, camera, thermal, satellite, or UAV observations, is also available.

The added value of bioindicator information should be tested rather than assumed. Future field studies should deploy instrumented hives alongside georeferenced conventional sensors, such as weather stations, particulate-matter or gas sensors, and fixed cameras or thermal sensors. Time-synchronized hive, environmental, and verified fire or smoke data could be used to compare conventional-sensor-only models with models that also include bee-derived features. Evaluation should assess whether bee-derived features improve detection lead time, false-alarm rate, alert prioritization, or verification efficiency under different wind, distance, terrain, seasonal, and non-fire disturbance conditions.

The proposed architecture has four layers. First, an environmental driver layer captures temperature, humidity, wind, fuel moisture, and air quality, the variables already discussed in Section 4.1 [1,129]. Second, a conventional sensing layer gathers data from satellite imagery, UAVs, and IoT/WSN nodes, processed using the AI methods described in Section 3.2 [45,139]. Third, a bee sensing layer consists of distributed hives with acoustic and environmental sensors, using lightweight AI models of the kind reviewed in Section 4.3 to classify colony state as normal, heat-stressed, or air-quality-stressed [76,203]. Fourth, a conceptual fusion layer would combine conventional wildfire-monitoring outputs, such as fire-weather indices or satellite-detected smoke, with bee-derived stress indicators to examine their value for local anomaly prioritization or verification. Fire-weather and IoT systems support wildfire-risk monitoring [111], while AI can analyze hive acoustic and environmental data for colony monitoring [204]. In this conceptual architecture, bee-derived signals are not treated as direct evidence of wildfire or as an independent pre-ignition warning. Instead, an abnormal hive response could be investigated as a local contextual signal when it occurs alongside physical evidence, such as elevated fire-weather risk, degraded air quality, camera-based smoke observations, satellite detections, or nearby sensor anomalies. The proposed fusion logic is a research hypothesis and has not yet been validated using wildfire-linked bee acoustic data. The proposed value of a bee-derived layer is not fire localization or replacement of conventional sensors. A hive has a known geographic location, but its acoustic signal cannot identify the origin or direction of a fire. Instead, hive data may provide an integrated biological response to local environmental disturbance. A single abnormal hive response would not be sufficient evidence of wildfire. However, if abnormal responses from one or more monitored hives occur together with elevated fire-weather conditions, degraded air quality, smoke observations, or local sensor anomalies, the system could prioritize verification by a camera, UAV, or fire-management operator. The added value of this approach should be tested against conventional sensor-only systems.

This hybrid approach offers potential opportunities because managed hives can provide continuous local observations where they are already maintained, particularly in areas with limited conventional sensor coverage [74,204]. However, important constraints remain. Bee responses depend on species, colony health, and management, and no existing dataset directly links bee acoustics to wildfire or smoke events, making calibration and validation a major open challenge [178,205]. Addressing this gap will require joint field deployments and multimodal datasets that pair wildfire variables with bee acoustic recordings before this approach can be operationally integrated into wildfire early-warning systems [139,184].

### 4.5. Towards Practical Bee-Based Wildfire Sensing

Realizing the hybrid architecture outlined above raises four practical challenges. First, no shared benchmark currently links wildfire datasets (e.g., satellite hotspot or smoke records) with bee bioacoustic datasets, making it impossible to evaluate how much a bee layer would add to early warning performance [139,203]. Second, most bee AI models are trained on a small number of colonies at one or two sites, raising open questions about generalization across bee species, hive designs, and regions [201,206]. Third, deployment at scale faces energy, connectivity, and data-governance constraints similar to those seen in wildfire IoT networks, compounded by the fact that hives are often privately owned, raising questions of data ownership and incentive [204,207]. Finally, there is currently no formal “sensor model” for bee colonies that specifies how acoustic or behavioral signals map onto specific environmental drivers such as heat or smoke, which will be needed before bee bioacoustics can be rigorously validated against wildfire risk [178,205]. Addressing these four challenges, through joint field datasets, cross-site validation, and closer collaboration between ecologists, beekeepers, and AI researchers, will be necessary before bee-based sensing can move from a conceptual outlook to an operational component of wildfire early-warning systems.

## 5. Conclusions and Limitations

This review examined AI-based wildfire monitoring across satellite and aerial remote sensing, UAVs, IoT/WSN ground sensing, and local or specialized systems. The evidence shows that no single sensing method or AI model is suitable for every wildfire-monitoring task. Satellite and aerial systems are most useful for regional surveillance, hotspot mapping, fire-risk estimation, and fire-spread monitoring. UAVs and fixed cameras are useful for local smoke and flame detection, hotspot localization, and visual verification. IoT/WSN systems are useful for continuous local monitoring and early warning in high-risk areas. Local systems support site-specific monitoring through fixed cameras, weather stations, air-quality sensors, acoustic devices, and local alert services.

AI model selection depends on the task, input data, and deployment setting. Lightweight detection models are most suitable for real-time camera and UAV monitoring. Segmentation models are useful when fire or smoke boundaries are needed. Spatio-temporal models are useful for fire-risk and fire-spread forecasting. Classical machine learning models remain useful for structured environmental data and low-power IoT/WSN systems. However, performance measures should be interpreted within their task and dataset context. Accuracy, F1-score, mAP, IoU, RMSE, latency, and energy use are not directly comparable across different wildfire tasks.

### 5.1. Cross-Domain Challenges and Limitations

The review identified major challenges that limit reliable wildfire monitoring across sensing domains. These challenges include limited data from different regions and fire regimes, class imbalance, few early-ignition and early-smoke samples, false alarms, limited cross-region and cross-sensor validation, energy and communication constraints, and limited uncertainty analysis. Many studies report strong results on local datasets, but fewer test whether models work across new regions, sensors, seasons, or fire conditions. Therefore, good results on one dataset should not be treated as evidence of reliable performance in all wildfire environments.

This review searched Scopus and IEEE Xplore, which provide strong coverage of AI, engineering, and remote-sensing research. However, studies indexed mainly in environmental, geoscience, or ecology-focused databases, such as Web of Science, ScienceDirect, or GeoRef, may have been missed. This limitation is particularly relevant to the exploratory bioindicator and bee-bioacoustic literature. Future reviews should include additional environmental and ecology-focused databases, especially when systematically reviewing bioindicator-based wildfire sensing.

### 5.2. Conditional Role of Bee Bioacoustics

This review also considered bee bioacoustics as an exploratory future direction. Bee colonies can respond to environmental stressors such as temperature, humidity, air quality, smoke exposure, and changes in floral resources. Hive acoustic and environmental signals can be monitored with low-cost sensors and analyzed using machine learning models. However, current bee studies mainly classify colony states, health conditions, or environmental-stress proxies. They do not validate direct wildfire detection, wildfire-smoke classification, earlier warning than conventional sensors, or ignition localization.

Bee-derived signals cannot replace georeferenced physical sensors, cameras, thermal sensing, UAV observations, or satellite data. A hive records conditions at the hive location, not the location of a fire. Hive response may also depend on wind direction, terrain, plume movement, source distance, colony condition, weather, disease, forage availability, and management activity. Therefore, bee anomalies should not be used as stand-alone wildfire alarms.

The possible value of bee bioacoustics should be tested in field studies. Future studies should deploy instrumented hives beside georeferenced weather stations, particulate-matter or gas sensors, fixed cameras, and thermal sensors. Researchers should collect time-synchronized hive acoustics, environmental data, smoke exposure data, and verified fire-event data. They should compare conventional-sensor-only models with models that also include bee-derived features. Bee bioacoustics should be considered a useful complementary signal only if it improves detection time, false-alarm rates, verification priority, or operational decision support.

### 5.3. Future Research

Looking ahead, we identify several priorities for future work:

- Collect multimodal datasets in fire-prone landscapes that include environmental variables, technical sensor outputs, and high-quality bee acoustic recordings before, during, and after fire seasons.
- Develop models that can run on edge devices at or near the hive, while also linking to cloud-based fusion frameworks for regional early-warning systems.
- Foster cooperation among ecologists, beekeepers, fire managers, and AI researchers to design experiments, validate indicators, and ensure that any deployment respects ecological and societal constraints.

With these steps, the synergy between AI technologies and bioindicator species like bees could support more sensitive, robust, and sustainable strategies for wildfire risk assessment in a warming and increasingly fire-prone world.

## Figures and Tables

**Table 1:** Search strings and initial number of retrieved records.

Stream	Database	Search String	Initial Number of Records
Wildfire sensing and AI	Scopus	TITLE-ABS-KEY ((“wildfire*” OR “forest fire*” OR “bushfire*”) AND (“detect*” OR “predict*” OR “early warning” OR “monitor*”) AND (“machine learning” OR “deep learning” OR “artificial intelligence” OR “AI” OR “IoT” OR “sensor*” OR “remote sensing” OR “UAV” OR “drone”))	5728
Wildfire sensing and AI	IEEE Xplore	((“wildfire” OR “forest fire” OR “bushfire”) AND (“detection” OR “prediction” OR “early warning” OR “monitoring”) AND (“machine learning” OR “deep learning” OR “AI” OR “IoT” OR “sensor” OR “UAV”))	1783

**Table 2:** Eligibility criteria for study selection.

Criterion	Inclusion	Exclusion
Literature type	Peer-reviewed journal articles and full conference papers	Reviews, books, book chapters, theses, posters, technical reports, and abstract-only records
Language	English	Non-English publications
Timeline	Published between 2019 and 2025	Published before 2019
Wildfire focus	Studies on wildfire or vegetation-fire detection, early warning, risk prediction, burned-area mapping, or environmental monitoring using satellite, aerial, UAV, IoT/WSN, or related sensing modalities	Studies focused only on post-fire management, suppression, ecological recovery, or socio-economic impacts without a detection, monitoring, or early-warning component
AI/data methods	Studies using machine learning, deep learning, statistical modeling, or advanced signal-processing methods relevant to wildfire sensing	Purely descriptive or conceptual papers without an implemented data-driven or analytical method

**Table 3:** Summary of quality assessment scores (mean per study, 0–2 scale).

Criterion	Description	Mean Score (0–2)
QA1	Problem definition and context	1.74
QA2	Data description	1.61
QA3	Methodological detail and reproducibility	1.73
QA4	Evaluation protocol and metrics	1.60
QA5	Limitations and threats to validity	1.34

**Table 4:** Cross-domain The synthesis of sensing modalities for AI-based wildfire monitoring.

Sensing Domain	Primary Operational Role	Main Strengths	Main Limitations	Most Suitable Wildfire Tasks
Satellite and aerial remote sensing	Regional and large-area surveillance	Wide spatial coverage, repeatable observations, and spectral and thermal information	Cloud and smoke occlusion, mixed pixels, revisit delays, and reduced sensitivity to small or short-duration fires	Regional active-fire monitoring, hotspot mapping, burned-area mapping, fire-risk estimation, and fire-spread monitoring [39,40,83,86,88]
UAV-based sensing	Local surveillance, tactical verification, and fire-front monitoring	High spatial resolution, flexible deployment, visible and thermal imaging, and close-range observation	Limited flight endurance, payload constraints, weather sensitivity, communication-range limits, operator workload, and regulatory constraints	Early visible smoke and flame detection, hotspot localization, fire-front tracking, and post-alert verification [55,91,92,93,94]
IoT and WSN ground sensing	Continuous local early warning in high-risk areas	Fine temporal sampling, local temperature, humidity, gas, smoke, and wind measurements, and low-latency edge or fog inference	Limited spatial coverage, dependence on node density, energy constraints, communication failures, and maintenance requirements	Environmental anomaly detection, local ignition warning, fire-weather monitoring, and sensor-triggered alerts [27,29,45,112,137]
Local and specialized systems	Site-specific continuous monitoring and operational decision support	Persistent observation through fixed cameras, weather stations, air-quality sensors, acoustic devices, and local communication systems	Limited field of view, local calibration needs, infrastructure dependence, and limited transferability across sites	Fixed-camera smoke and flame detection, local air-quality monitoring, embedded audio detection, and multi-source alerting [18,72,124,125,135]

**Table 5:** Summary of AI model categories across wildfire detection tasks and sensing domains.

Model Category	Typical Models	Sensing Context	Key Observations
Classical Machine Learning (Risk and Prediction)	Random Forest, SVM, regression models	Satellite data, environmental and climatic variables	Effective for fire occurrence and spread prediction; interpretable but sensitive to spatial and temporal domain shifts
CNN-based Detection (Fire/Smoke)	CNN, FireNet, 3D-CNN	CCTV cameras, UAV imagery, satellite images	High classification accuracy (>90%); widely used for real-time fire and smoke detection
Segmentation Models (Burned Area and Smoke)	U-Net, encoder–decoder CNNs	Satellite imagery, UAV data	Improved spatial localization and boundary detection; outperform threshold-based approaches
Object Detection (Small Targets)	YOLO variants, Faster R-CNN, Transformer-based detectors	UAV, CCTV, industrial and remote sensing imagery	Strong performance for small and early-stage fire/smoke detection; suitable for real-time deployment
Spatio-Temporal Models (Forecasting)	Deep learning, time-series models, hybrid ML	Multi-source environmental and climate data	Capture temporal dynamics and environmental dependencies; useful for early warning but sensitive to data quality
Edge/Embedded AI	Pruned CNNs, TinyML models	IoT devices, edge cameras, embedded platforms	Enables low-latency and energy-efficient detection; constrained by hardware and model size
Federated/Distributed Learning	Federated learning frameworks, optimization-based aggregation	Distributed IoT and multi-region datasets	Preserves data privacy and reduces communication cost; introduces challenges in heterogeneity and robustness

**Table 6:** Evidence matrix for generalization, transferability, data limitations, fusion, deployment, and uncertainty in AI-based wildfire monitoring.

Domain	Cross-Region Generalization	Cross-Sensor Transfer	Data Scarcity/Imbalance	Multimodal Fusion	Real-Time/Edge Constraints	Uncertainty Quantification
Satellite and aerial	Partially reported. EO4WildFires covers 45 countries and Sen2Fire uses geographic splits; many studies remain region specific.	Partially reported. Multi-satellite fusion is used, but transfer across satellite, UAV, and ground sensors is rarely evaluated.	Partially reported. Augmentation and generative methods reduce annotation needs, but early-smoke and small-fire samples remain scarce.	Evidence reported. Multi-satellite products are fused for detection, mapping, and fire-radiative- power estimation.	Rarely reported. Near-real-time products exist, but revisit delay and atmospheric conditions limit early detection; edge deployment is uncommon.	Rarely reported. Uncertainty-aware forecasting exists, but satellite-specific evaluation is uncommon.
UAV-based	Rarely reported. Most studies use local image datasets; cross-region held-out testing is uncommon.	Partially reported. Visible–thermal fusion is used, but transfer across UAV, satellite, and IoT data is rarely tested.	Partially reported. Lightweight and small-target models are used, but scarce early-ignition samples remain under-evaluated.	Evidence reported. Visible, infrared, thermal, and IoT-connected UAV sensing are combined for detection and localization.	Frequently reported. Lightweight models support onboard or near-real-time inference, but endurance, communication, weather, and safety remain constraints.	Rarely reported. Formal uncertainty analysis is uncommon.
IoT and WSN ground sensing	**Rarely reported.** Prototype, simulation, and single-site evaluations dominate.	**Rarely reported.** Transfer across sensor types and configurations is seldom tested.	**Partially reported.** Some models tolerate noise and missing data, but scarce real-fire data and imbalance remain challenges.	**Evidence reported.** Sensor, weather, camera, UAV, and cloud data are combined in several systems.	**Frequently reported.** Edge and fog systems reduce latency, but energy, network reliability, and maintenance remain constraints.	**Rarely reported.** Formal uncertainty analysis is uncommon.
Local and specialized systems	**Rarely reported.** Fixed-camera and audio systems are usually tested at one or few sites.	**Partially reported.** Camera, weather, air-quality, audio, and social-media streams are combined, but transfer across sites is rarely tested.	**Rarely reported.** Dedicated methods for scarce early-fire or early-smoke samples are uncommon.	**Evidence reported.** Local architectures combine camera, weather, air-quality, audio, and alert-service data.	**Evidence reported.** Embedded image and audio systems support real-time operation under hardware and power constraints.	**Rarely reported.** Formal uncertainty analysis is uncommon.
Federated and robustness studies	**Partially reported.** Federated approaches support training across distributed clients or regions without centralizing raw data.	Not applicable	**Partially reported.** Distributed data can improve diversity, but labeled data remain necessary.	Not applicable	**Partially reported.** Communication cost can be reduced, but device heterogeneity and client participation remain challenges.	**Evidence reported.** A small number of studies explicitly model uncertainty, robustness, or distribution shift.

Note: “Evidence reported” indicates that one or more included studies explicitly evaluated the issue. “Partially reported” indicates that relevant approaches or limited examples were identified, but evaluation was incomplete or restricted to specific datasets, sites, or sensor types. “Frequently reported” indicates that the issue was addressed in several studies within the domain. “Rarely reported” indicates that few included studies explicitly evaluated the issue. These labels describe reporting patterns in the reviewed literature and are not formal certainty-of-evidence ratings.

**Table 7:** Comparison of multimodal fusion strategies reported in AI-based wildfire monitoring, including deployment trade-offs.

Fusion Strategy	Integration Level	Reported Benefit	Reported Limitation	Representative Studies and Findings
Early or input-level fusion	Raw or preprocessed environmental, satellite, or sensor variables are combined before model learning	Combines complementary spatial, temporal, and environmental information for fire-risk prediction	Requires compatible spatial and temporal resolution; performance remains dependent on data coverage and local conditions	Multimodal time-series wildfire prediction [66]; multi-sensor five-day early prediction with model accuracy above 80% [123].
Intermediate or feature-level fusion	Modality-specific features are extracted and merged through concatenation, attention, or cross-modal modules	Can improve representation of smoke, flames, and small targets under variable illumination and complex backgrounds	Higher model complexity; requires aligned sensor inputs and is rarely tested across sites	Visible–infrared UAV fusion for early detection [97]; RGB–thermal target-aware feature fusion using cross-modal interaction and attention [43].
Decision-level fusion	Independent model outputs are combined through weighted voting, rules, or decision thresholds	Combines visual fire evidence with independent environmental-risk estimates	Few studies evaluate modality ablation, missing sensor streams, or transfer to new sensing configurations	Weighted decision-level fusion of ViT–GRU visual classifications and LSTM–Optuna meteorological fire-risk predictions [64].
System-level orchestration	One sensing component detects, triggers, verifies, or prioritizes another component without direct feature merging	Supports staged monitoring, alarm verification, and targeted data collection across distributed systems	Depends on communication reliability, sensor coverage, alert thresholds, power availability, and operational coordination	Camera, meteorological, and satellite data combined in SmokeyNet [138]; IoT sensor flags collected and verified by patrolling UAVs [100].

Note: The reported findings come from different tasks, datasets, sensing configurations, and validation methods. Therefore, they are not directly comparable across studies. Few studies test the effect of removing one sensing modality, sensor failure, or differences between fusion strategies.

**Table 8:** Normal and deviant bee behaviors under environmental and climatic stressors reported in the reviewed literature.

Behavior Aspect	Normal Behavior	Deviated Behavior Under Stress	Key Evidence	References
Foraging activity	Active foraging at 21–33.5 °C; peak activity in morning/early afternoon	Foraging declines above 33.5 °C and nearly stops above 43 °C; poor air quality increases trip duration	Pollution increased foraging trip duration by 32 min	[75,177,179,185,186]
Colony temperature regulation	Brood nest maintained at 33–35 °C through fanning and water collection	Heat stress causes brood temperature fluctuation, dehydration, and increased fanning	Fanning increased up to 300% at 40 °C	[177,179]
Brood rearing	Brood rearing continues under mild climate and sufficient resources	Heat stress and poor resources reduce brood rearing and increase disease/ Varroa susceptibility	Brood rearing decreases during heatwaves; Varroa risk increases with longer brood periods	[43,180,187]
Flight activity timing	Flight activity peaks around 9 AM–2 PM and varies with floral availability	Extreme heat or smoke exposure can reduce or shift flight activity	Flight timing varies by plant species and environmental conditions	[185,188]
Olfactory sensitivity	Strong antennal sensitivity supports floral scent detection, learning, and memory	Heatwaves, ozone, and pollutants reduce scent response and impair olfactory learning	Olfactory response decreased up to 80% after heatwaves	[189,190,191]
Communication and acoustic signals	Stable waggle dance, piping, and acoustic communication under normal conditions	Smoke and stress disrupt signals, increase stop signals, and alter dance accuracy	Stop signals increased 4x during smoke; distress piping reported at 250–280 Hz	[184,192]
Temperature stress	Brood comb usually maintained at 33–36 °C; bees regulate hive temperature by fanning, clustering, and water collection	Foraging decreases above 35 °C and ceases above 43 °C; heat stress causes dehydration, reduced brood, and altered movement	CTmax: honeybees ∼49.1 °C; bumblebees ∼53.1 °C; sweat bees ∼50.3 °C	[177,179,187,193,194]
Humidity stress	Hive RH is commonly maintained around 50–75%; brood humidity is optimal at 90–95% for egg hatching	Very low RH (<30%) or high RH (>75%) disrupts hygroregulation and increases metabolic stress	Best worker survival reported at 75% RH; survival drops at 15% RH; brood hatching best at 90–95% RH	[4,74,177,181,195,196]
CO_2_ stress	Typical in-hive CO_2_ is higher than ambient air; bees tolerate moderate hive CO_2_ levels and increase fanning as CO_2_ rises	High acute CO_2_ exposure can alter queen physiology and colony metabolism; chronic CO_2_ may indirectly affect nutrition through pollen quality	Daily in-hive CO_2_ range reported as 0.33–1.77%; no mass mortality observed up to 1.77%	[76,197,198,199,200]

## Data Availability

All data analyzed in this study are from previously published articles cited in this review. Any additional data generated during the synthesis, such as screening records and summary tables, are available from the corresponding author on request. The review registration record is available on OSF (https://doi.org/10.17605/OSF.IO/VH7KE).

## Abbreviations

The following abbreviations are used in this manuscript:

AI	Artificial Intelligence
AP	Average Precision
AR	Autoregressive
AUC	Area Under the Curve
CCTV	Closed-Circuit Television
CNN	Convolutional Neural Network
CO_2_	Carbon Dioxide
DL	Deep Learning
EO4WildFires	Earth Observation for Wildfires
F1	F1-score
FedAvg	Federated Averaging
FLOPs	Floating Point Operations
FWI	Fire Weather Index
GPU	Graphics Processing Unit
IEEE	Institute of Electrical and Electronics Engineers
IoT	Internet of Things
IoU	Intersection over Union
IR	Infrared
LoRa	Long Range
LSNet	Lightweight Segmentation Network
MAE	Mean Absolute Error
mAP	mean Average Precision
MCU	Microcontroller Unit
ML	Machine Learning
NASA POWER	NASA Prediction of Worldwide Energy Resources
NB-IoT	Narrowband Internet of Things
PDAM–STPNNet	Pixel-Level Dense Attention Module–Spatio-Temporal Prediction Network
PM_2.5_	Particulate Matter with Diameter of 2.5 Micrometers or Less
PRISMA	Preferred Reporting Items for Systematic Reviews and Meta-Analyses
PSO	Particle Swarm Optimization
QA	Quality Assessment
RGB	Red–Green–Blue
RH	Relative Humidity
RMSE	Root Mean Square Error
RQ	Research Question
SAR	Synthetic Aperture Radar
SLR	Systematic Literature Review
SVM	Support Vector Machine
TinyML	Tiny Machine Learning
UAV	Unmanned Aerial Vehicle
WSN	Wireless Sensor Network
XAI	Explainable Artificial Intelligence
YOLO	You Only Look Once

## Appendix A. Representative AI Models and Metrics Across Tasks and Domains

**Table A1:** Representative AI models and metrics across tasks and domains (RQ2)—Part I.

Model and Key Idea	Sensing Domain and Data	Deployment Profile	Main Reported Metrics/Takeaway
**Classical ML—satellite risk and arrival-time modeling**			
ML framework for fire arrival-time estimation [147,150,170]	Satellite active-fire products; Level 2 active-fire detections with ancillary variables	Offline forecasting support	Lower RMSE than physical extrapolation baselines
Spatial-scale ML for wildfire drivers [6,123,148,151]	Remote sensing and environmental covariates; regional fire occurrence and driver datasets	Offline risk mapping	Identifies predictive drivers and suitable spatial scales
**Classical ML—integrated IoT/WSN early warning detection**			
WSN + deep learning early fire detection [16,29]	IoT/WSN ground sensing; temperature, humidity, and smoke sensors	Edge/gateway	High detection rate; fewer false alarms than rule-based WSN
Sensor and DNN wildfire prediction [47,208,209]	IoT environmental sensor network data	Edge + cloud	Higher prediction accuracy than threshold methods
Integrated WSN + ML detection system [64,154,155]	IoT/WSN prototype sensor readings	Edge/gateway	Reliable detection with modest energy and computation cost
**CNN classifiers—camera-based fire/smoke detection**			
FireNet-style CNN classifier [140,141]	CCTV/local cameras; curated fire vs. non-fire images	Server/cloud; near-real time	Accuracy and F1 commonly above 90%
3D parallel fully CNN for video smoke detection [210]	CCTV/local cameras; labeled smoke/non-smoke video frames	GPU server; real-time	High frame-level accuracy at video rate
CNN classifier for satellite wildfire images [211,212]	Satellite optical imagery; regional fire/non-fire satellite scenes	Offline/cloud	Accuracy and recall above classical ML baselines
CNN integrated with IoT/WSN triggers [70,156,208]	IoT sensors and fixed cameras; sensor-triggered image data	Edge/gateway + cloud	High image-level accuracy; fewer false alarms than sensors alone
**Encoder–decoder CNNs—burned-area and smoke segmentation**			
U-Net-based burned-area mapping [20,54]	Satellite optical imagery; pre- and post-fire images with burn masks	Offline regional mapping	Higher IoU and F1 than index-threshold methods
LSNet with attentional fusion [67]	CCTV/local cameras; pixel-labeled wildfire image dataset	Edge/GPU; real-time capable	High IoU with low latency
Optimized U-Net for smoke segmentation [146,213]	CCTV/UAV imagery; annotated smoke masks in natural scenes	Server/edge GPU	Improved IoU and boundary quality vs. vanilla U-Net

**Table A2:** Representative AI models and metrics across tasks and domains (RQ2)—Part II.

Model and Key Idea	Sensing Domain and Data	Deployment Profile	Main Reported Metrics/Takeaway
**Object detection and small targets—YOLO, transformer, and attention-based models**			
Improved YOLOv5 for UAV smoke detection [55,93,96]	UAV RGB imagery; smoke images with bounding boxes	Onboard GPU/edge; real-time	mAP and F1 above 90%; robust to small smoke plumes
Lightweight YOLOv8 variants, including FFYOLO [96,164,214]	UAV/CCTV images; fire and smoke boxes	Edge/server	Higher mAP and small-object AP than base YOLOv8; fewer parameters
Fire-YOLO for small fire targets [143,145]	CCTV/industrial scenes; small fire target data	Edge/cloud	Improved precision and recall for tiny fire objects
PDAM–STPNNet with attention [142]	Satellite remote sensing; small smoke target images	Cloud/offline	Higher small-object detection rate than standard CNN detectors
Lightweight YOLOv11-style detector [145,214]	CCTV/smart factory scenes; fire and smoke data	Edge GPU	High mAP with reduced computation; suitable for embedded use
Faster R-CNN with Swin Transformer [51]	CCTV/multiclass wildfire imagery; fire, smoke and non-fire images	GPU server	Higher mAP and robustness than vanilla Faster R-CNN
Transformer-enhanced UAV fire detection/segmentation [52,167]	UAV RGB imagery; drone fire labels or masks	Edge GPU/ground station	Improved precision–recall and multiscale handling vs. pure CNNs
**Spatio-temporal ML—occurrence and spread forecasting**			
Spatio-temporal prediction of steppe fires [6,148,215]	Remote sensing, weather and environmental covariates; historical fire records	Offline forecasting	Good skill for next-day fire occurrence patterns
Deep learning for regional wildfire risk [149,170,212,216]	Multimodal climate and environmental data; climate, fuel, topography and fire history	Cloud/offline	Improved AUC and RMSE compared with classical ML baselines
Early prediction using multisensor data and ML [123,217,218]	Multi-source remote sensing and environmental fire-related sensor data	Cloud/offline	High predictive accuracy with feature-importance and uncertainty analysis
Recursive transformer for multi-source fusion [22,66,67]	Satellite and ancillary data; remote sensing and environmental time series	Cloud; near real time	Around 10 min early detection horizon with strong accuracy
Trend-based wildfire detection under AR errors [150,151]	Climate and fire-indicator time series	Offline statistical forecasting	Reduced detection error compared with simpler trend methods

**Table A3:** Representative AI models and metrics across tasks and domains (RQ2)—Part III.

Model and Key Idea	Sensing Domain and Data	Deployment Profile	Main Reported Metrics/Takeaway
**Edge/embedded CNNs—low-power deployment**			
Fourier-pruned CNN for efficient fire detection [59,141,163]	CCTV/local cameras; fire vs. non-fire video data	Edge GPU/embedded; real-time	High accuracy with reduced FLOPs and parameters
Embedded audio–image wildfire detection [48]	Local microphones and cameras; custom audio–image wildfire data	TinyML/MCU devices	Real-time response under strict memory and power limits
Low-power environmental monitoring with on-device AI [28,46,114,116]	IoT/WSN environmental sensor platform data	Edge/gateway	Maintains detection while reducing energy use and data transmission
Fog/edge ML classifier for early detection [27,213]	IoT/WSN; synthetic and real sensor datasets	Fog/edge servers	Higher accuracy and lower latency than cloud-only processing
Sensor-based AI in social IoT wildfire framework [121,122,219]	IoT sensors, environmental data and social/user platform inputs	Edge + cloud	Timely alerts and improved situational awareness
**Federated learning—distributed detection and prediction**			
PSO-optimized federated learning for fire detection [71,171]	Multi-client fire image data; forest fire images partitioned by client	Federated clients	Mid-90% accuracy with reduced communication vs. FedAvg
Federated wildfire area prediction [171]	Multi-region climate and fire data; regional environmental datasets	Federated; cross-region	Comparable or better RMSE/MAE than centralized training
Robustness analysis for wildfire prediction models [152]	Benchmark wildfire prediction datasets and models	Design time; cloud/edge/federated	Shows sensitivity to adversarial perturbations; supports robust training

## Appendix B. Exploratory Bee-Based Monitoring Evidence Relevant to Wildfire Monitoring

**Table A4:** Structured exploratory evidence from bee-based monitoring studies and its current relationship to wildfire monitoring. These studies are not part of the PRISMA-screened 169-study wildfire corpus.

Area of Study/Study	Signal Measured	Stressor or Target Condition	Dataset/Setting	AI Method	Real Smoke/Fire Condition?	Wildfire Relevance and Limitation
**AI-based hive state and health monitoring**						
Bee colony anomaly detection [220]	Hive audio	Normal, queenless, virus-infected, and pesticide-contaminated colonies	IoT audio monitoring and field experiments with managed hives	SVD features with KNN, LR, and SVM; edge classification	No	No wildfire validation. Demonstrates AI classification of colony anomalies and stress proxies.
Queen-status monitoring [221]	Hive audio and spectral features	Queen presence or absence	Honeybee audio datasets with normal and queen- missing colonies	SVM and neural- network classifiers	No	No wildfire validation. Supports acoustic classification of a biologically meaningful colony state.
**AI-based hive state and health monitoring**						
Swarm prediction [222]	Hive audio, waveplots, Mel spectrograms, and MFCCs	Swarm versus normal colony activity	NU-Hive recordings from hives in Europe, North America, and Australia	Naive Bayes, KNN, SVM, CNN, LSTM, and Transformer models	No	No wildfire validation. Shows AI classification of swarm-related acoustic patterns.
Multisensor colony-health forecasting [201]	In-hive sensor, weather, and inspection data	Colony health status and short-term hive conditions	Managed-hive sensor, weather, and inspection records	Machine learning forecasting model	No	No wildfire validation. Demonstrates combined hive and weather data for colony monitoring.
Varroa-risk monitoring [223]	Temperature, humidity, CO_2_, and TVOC	Varroa infestation risk	IoT prototype with sensor data and synthetic classification data	Supervised machine- learning classifiers	No	No wildfire validation. Shows environmental hive sensing for health-risk classification.
**Bee acoustics and environmental stress**						
Wingbeat sound and induced stress [37]	Wingbeat frequency, duration, energy, and MFCC features	Air temperature and experimentally induced stress	Field recordings of domestic and wild bees in Spain	Random Forest classifier and mixed-effects modeling	No	Indirect environmental evidence. Acoustic features vary with temperature and induced stress, but wildfire and smoke specificity remain untested.
Multimodal bumblebee field dataset [224]	Acoustic recordings, temperature, humidity, pressure, CO_2_, and gas measurements	Native and invasive bumblebee activity under field environmental conditions	Field dataset from 12 locations in Patagonia over six days	Dataset designed for future machine learning classification	No	Indirect ecological- monitoring evidence. Provides synchronized acoustic and environmental data but contains no wildfire or smoke labels.
Queen-status monitoring [221]	Hive audio and spectral features	Queen presence or absence	Honeybee audio datasets with normal and queen- missing colonies	SVM and neural-network classifiers	No	No wildfire validation. Supports acoustic classification of a biologically meaningful colony state.
**Air-quality and fire-ecology evidence**						
Air quality and colony mortality [225]	Honeybee colony mortality records with air-quality, weather, and vegetation variables	Poor air quality, ozone, Air Quality Health Index, and vegetation availability	Longitudinal data from more than 103,000 managed hives in Canada and the United States	Generalized linear mixed models and Random Survival Forest	No direct smoke-exposure experiment; wildfire-related air-quality context	Indirect wildfire-related evidence. Poor air quality predicts colony mortality, but the study does not use hive acoustics or detect wildfire events.
Bee responses to fire regimes [226]	Bee abundance, diversity, nesting, behavior, and life-history responses	Fire disturbance, prescribed burning, wildfire, and post- fire conditions	Review of bee–fire ecology studies	No AI method	Yes—fire-related ecological evidence	Direct fire-ecology evidence. Fire affects bee communities and habitats, but no acoustic AI wildfire-detection model is reported.

Existing evidence supports colony-state classification, environmental-stress monitoring, air-quality effects, and bee fire ecology. However, no identified study validates direct wildfire detection, labeled wildfire-smoke classification, earlier detection than conventional sensors, or ignition localization from bee acoustic signals.

The evidence in Table A4 indicates that bee-based AI studies currently support colony-state and environmental-stress monitoring rather than direct wildfire detection. The table is included as an exploratory evidence summary and should not be interpreted as validation of bee acoustics for wildfire-smoke detection, earlier warning, or ignition localization.
